# Supplementary material for: Plekhg5 controls the unconventional secretion of Sod1 by presynaptic secretory autophagy
Source: Nat Commun. 2024 Oct 4;15:8622. doi: 10.1038/s41467-024-52875-5 (PMC11452647; doi:10.1038/s41467-024-52875-5)
Supplement: Supplementary file 1 — Supplementary Information [file 41467_2024_52875_MOESM1_ESM.pdf]

Supplementary Information

Plekhg5 controls the unconventional secretion of Sod1 by presynaptic secretory autophagy

Amy-Jayne Hutchings, Bita Hambrecht, Alexander Veh, Neha Jadhav Giridhar, Abdolhossein Zare, Christina Angerer, Thorben Ohnesorge, Maren Schenke, Bhuvaneish T. Selvaraj, Siddharthan Chandran, Jared Sterneckert, Susanne Petri, Bettina Seeger, Michael Briese, Christian Stigloher, Thorsten Bischler, Andreas Hermann, Markus Damme, Michael Sendtner, Patrick Lüningschrör

Content:

Supplementary Figures 1-4
Supplementary Table 1: Cell lines
Supplementary Table 2: Mouse strains
Supplementary Table 3: Plasmids
Supplementary Table 4: Primary antibodies for immunohistochemistry
Supplementary Table 5: Primary antibodies for Western blot
Supplementary Table 6: Oligonucleotides

Supplementary Figures

Supplementary Figure 1

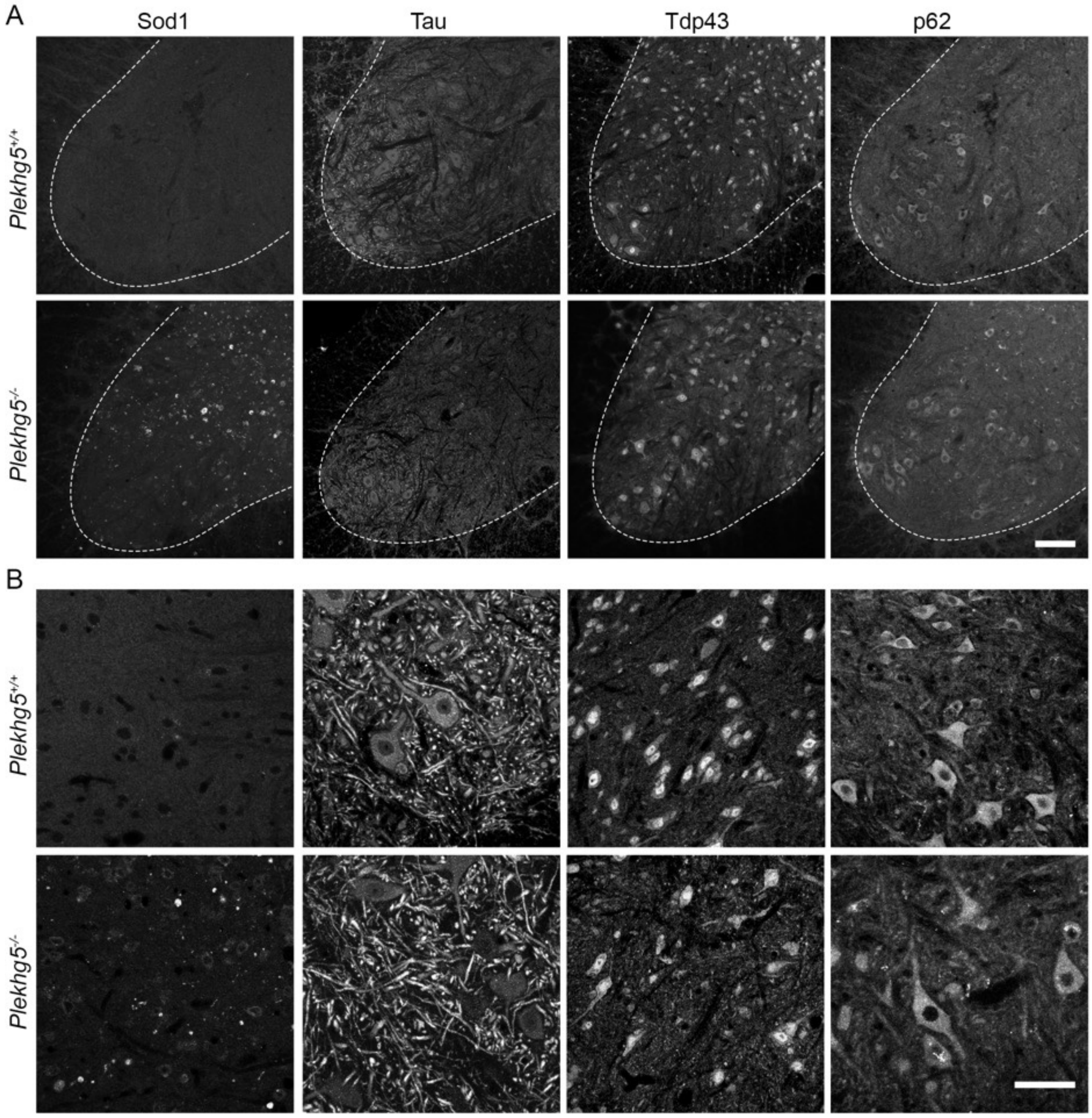

Plekhg5 deficiency does not result in a general accumulation of aggregation-prone proteins. (A, B) Lumbar spinal cord cross sections from wildtype and Plekhg5-deficient mice stained for Sod1, Tau, Tdp43 and p62. Images of the ventral horn are shown in (A). Higher magnifications are shown in (B). Note that Plekhg5 deficiency only caused accumulations of Sod1. Scale bar upper panel: 100 µm; Scale bar lower panel: 50 µm. The images are representative of at least three biological replicates.

Supplementary Figure 2

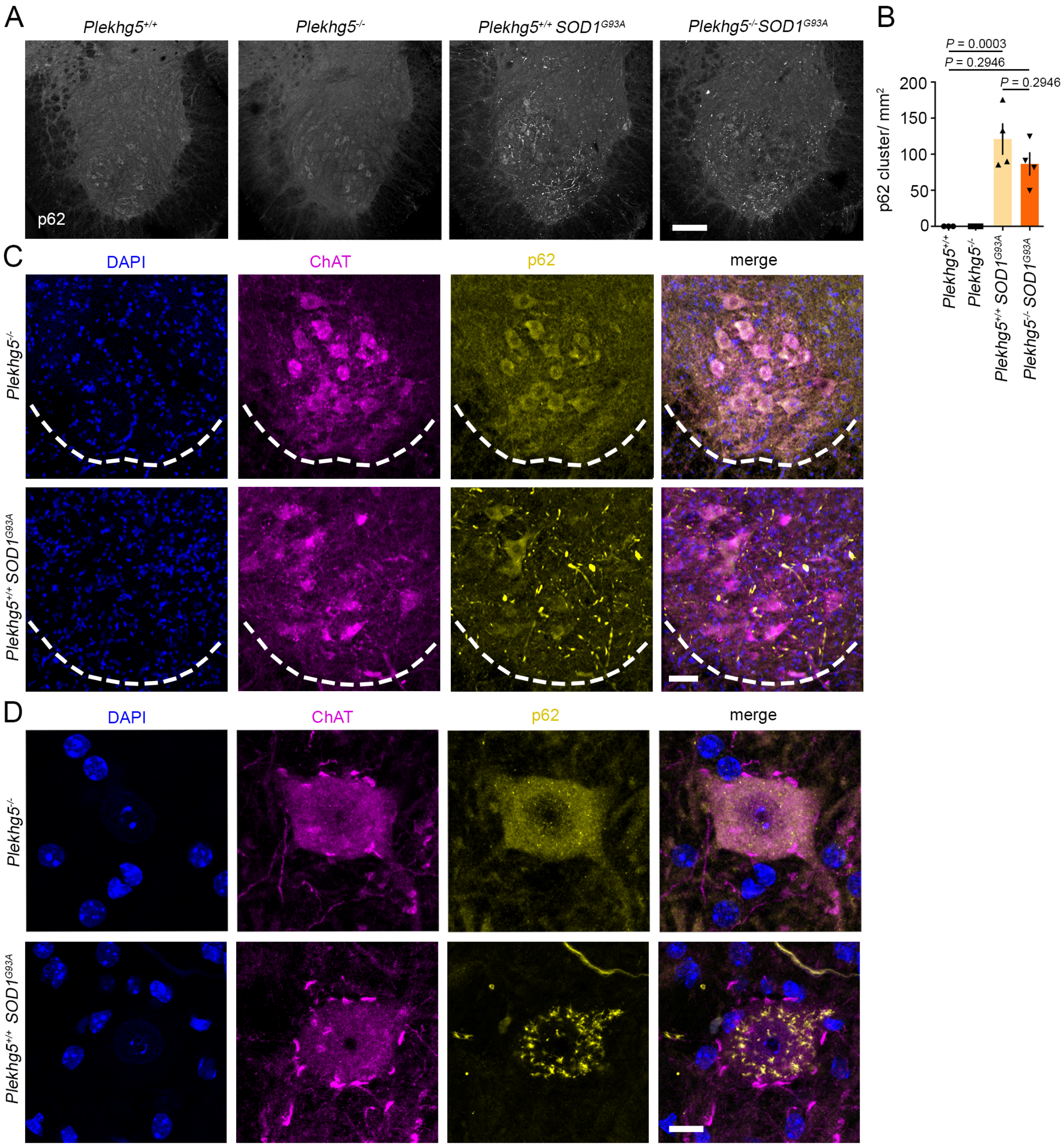

Plekhg5 depletion in SOD1^{G93A} mice does not affect the accumulation of p62 within the spinal cord. (A) Spinal cord cross sections stained for p62. Depletion of Plekhg5 did not cause any differences in the number of p62 clusters in SOD1^{G93A}-expressing mice. The images are representative of at least three biological replicates. Scale bar: 200 µm. **(B)** Quantification of p62 clusters. Mean±SEM. One-way ANOVA; Tukey's Multiple Comparisons. *Plekhg5*$^{+/+}$, n=3; *Plekhg5*$^{-/-}$, n=4; *Plekhg5*$^{+/+}$ *Sod1*^{G93A}, n=4; *Plekhg5*$^{-/-}$ *Sod1*^{G93A}, n=4; biological replicates. Mean±SEM. **(C, D)** Spinal cord cross sections co-stained for p62 and ChAT (C). High magnification images of an individual spinal motoneuron (D). p62 clusters were absent in Plekhg5-deficient mice, and only detectable in SOD1^{G93A}-expressing mice. The images are representative of at least three biological replicates. (C) Scale bar: 50 µm. (D) Scale bar: 10 µm.

Supplementary Figure 3

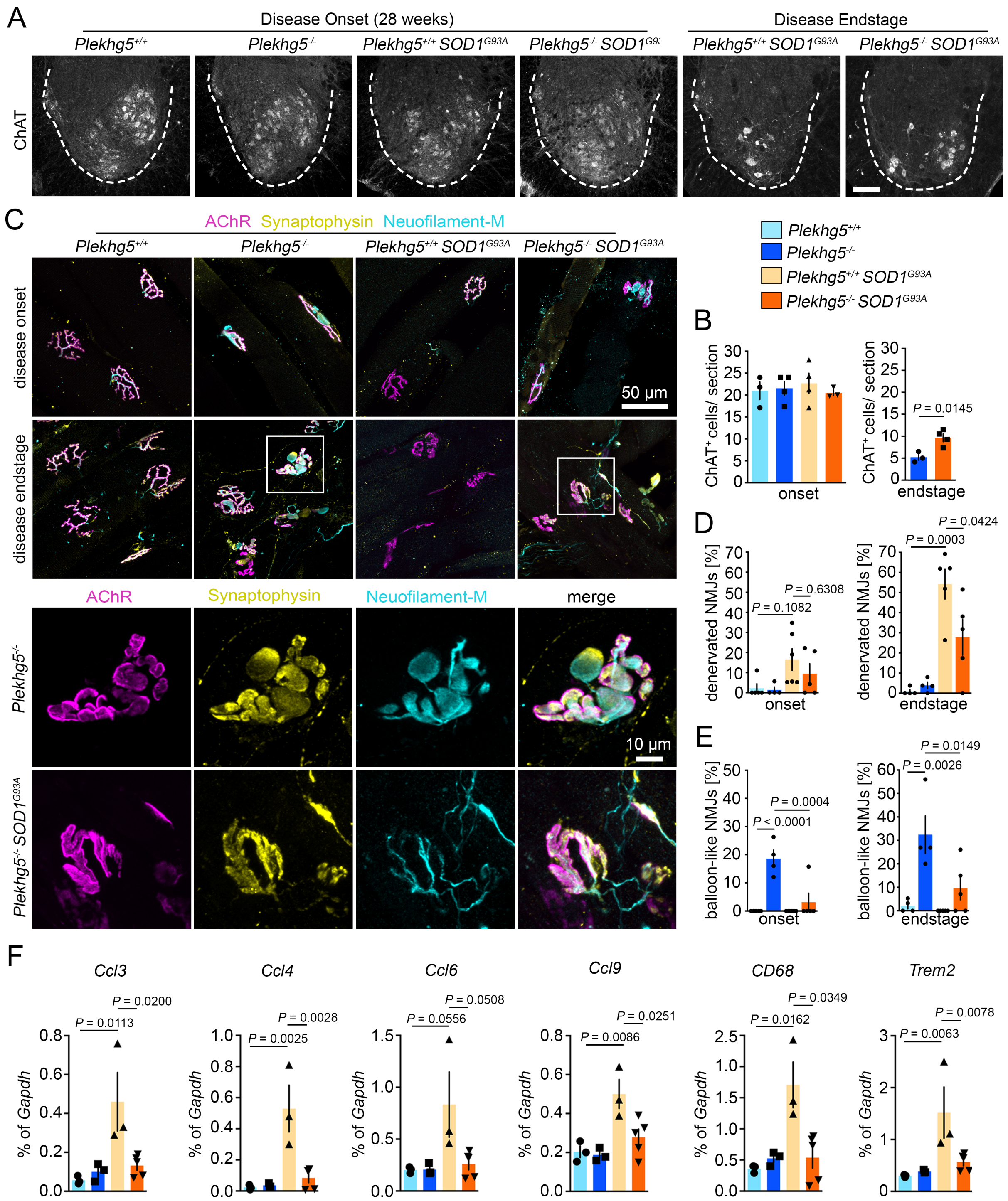

Motoneuron quantification and NMJ characterization. (A) ChAT staining in spinal cord sections of *Plekhg5*$^{+/+}$, *Plekhg5*$^{-/-}$, *Plekhg5*$^{+/+}$ *Sod1*^{G93A} and *Plekhg5*$^{-/-}$ *Sod1*^{G93A} mice at disease onset and disease endstage show reduced ChAT levels in *Plekhg5*$^{+/+}$ *Sod1*^{G93A} and *Plekhg5*$^{-/-}$ *Sod1*^{G93A} mice at disease endstage. Scale bar: 200 µm. The images are representative of at least three biological replicates. **(B)** Quantification of ChAT^{+} cells. MN survival in *Plekhg5*$^{-/-}$ *Sod1*^{G93A} mice was slightly, but significantly improved at the disease endstage compared to *Plekhg5*$^{+/+}$ *SOD1*^{G93A} mice. t-test, two-tailed. Onset: *Plekhg5*$^{+/+}$, n=3; *Plekhg5*$^{-/-}$, n=4; *Plekhg5*$^{+/+}$ *Sod1*^{G93A}, n=4; *Plekhg5*$^{-/-}$ *Sod1*^{G93A}, n=3. Endstage:

Plekhg5$^{+/+}$ *Sod1*^{G93A}, n=3; *Plekhg5*$^{-/-}$ *Sod1*^{G93A}, n=4; biological replicates. **(C)** NMJs visualized by BTX, synaptophysin and NF-M staining in *Plekhg5*$^{+/+}$, *Plekhg5*$^{-/-}$, *Plekhg5*$^{+/+}$ *Sod1*^{G93A} and *Plekhg5*$^{-/-}$ *Sod1*^{G93A} mice. Note that balloon-like NMJs are only present in *Plekhg5*$^{-/-}$ and *Plekhg5*$^{-/-}$ *Sod1*^{G93A} mice. The images are representative of at least three biological replicates. **(D, E)** Quantification of denervated NMJs and balloon-like NMJs at the disease onset (D) and endstage (E). NMJ denervation is significantly increased in *Plekhg5*$^{+/+}$ *Sod1*^{G93A} compared to *Plekhg5*$^{-/-}$ and *Plekhg5*$^{-/-}$ *Sod1*^{G93A} mice at disease onset and endstage. Balloon-like NMJs structures in *Plekhg5*$^{-/-}$ and *Plekhg5*$^{-/-}$ *Sod1*^{G93A} mice increase at disease endstage but do not coincide with NMJ denervation. One-way ANOVA; Tukey's Multiple Comparisons. Onset: *Plekhg5*$^{+/+}$, n=5; *Plekhg5*$^{-/-}$, n=4; *Plekhg5*$^{+/+}$ *Sod1*^{G93A}, n=6; *Plekhg5*$^{-/-}$ *Sod1*^{G93A}, n=5. Endstage: *Plekhg5*$^{+/+}$, n=4; *Plekhg5*$^{-/-}$, n=4; *Plekhg5*$^{+/+}$ *Sod1*^{G93A}, n=5; *Plekhg5*$^{-/-}$ *Sod1*^{G93A}, n=5; biological replicates. **(F)** Reduced microglia activation *Plekhg5*$^{-/-}$ *Sod1*^{G93A}. Expression of selected transcripts associated with microglia activation were analyzed by qPCR. *Plekhg5*$^{+/+}$, n=3; *Plekhg5*$^{-/-}$, n=3; *Plekhg5*$^{+/+}$ *Sod1*^{G93A}, n=3; *Plekhg5*$^{-/-}$ *Sod1*^{G93A}, n=5; biological replicates. One-way ANOVA; Tukey's Multiple Comparisons.

All data are shown as Mean±SEM.

Supplementary Figure 4

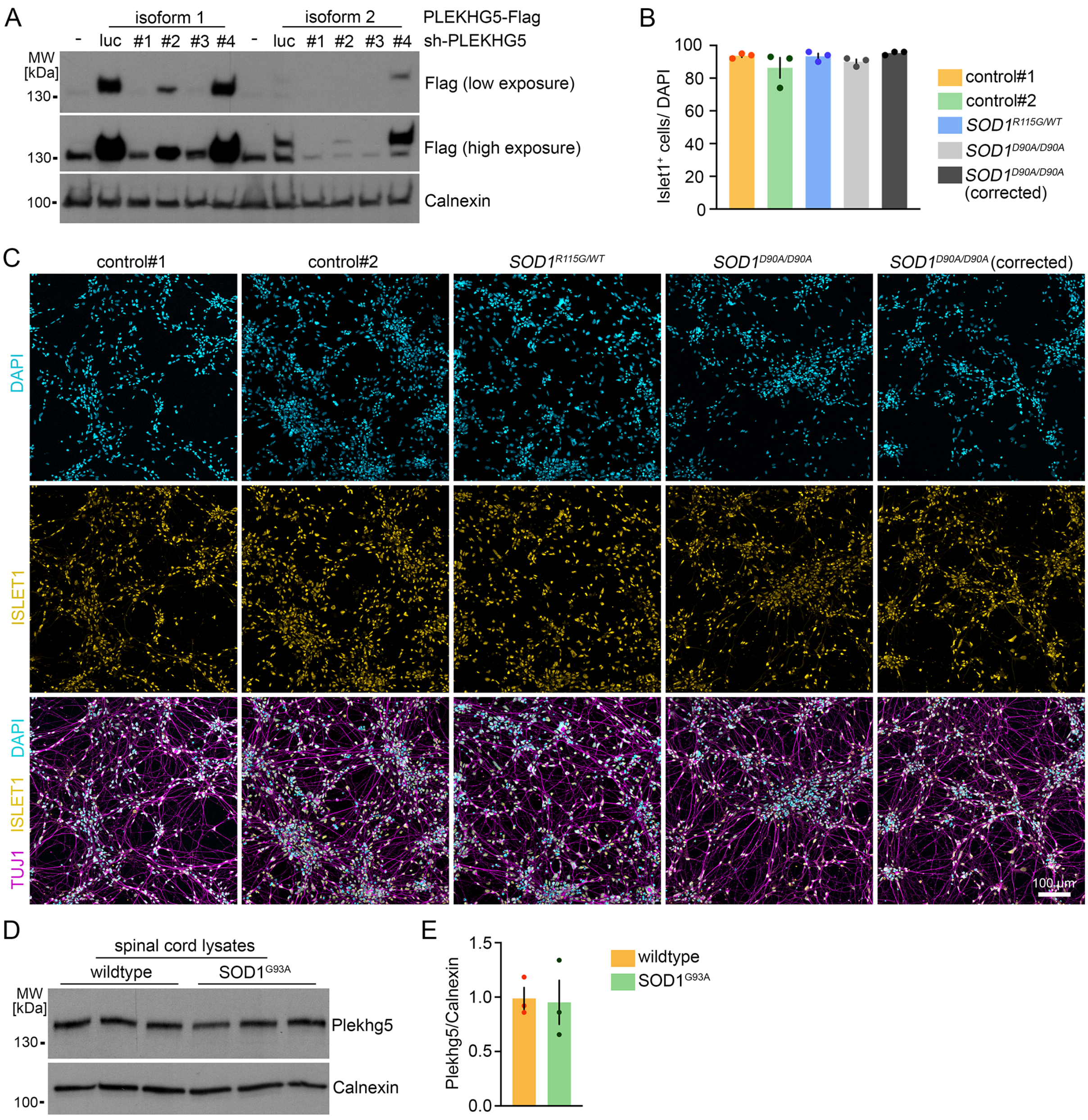

Secretion of SOD1 in hiPSC-derived MNs. (A) Four different sh-sequences were tested for their efficiency to knockdown PLEKHG5. Flag-PLEKHG5 Isoform 1 and Isoform 2 were transfected into 293FT cells along the indicated plasmids for expression of the individual sh-RNAs. **(B)** Quantification of the percentage of Islet1^{+} cells per nuclei. Each data point represents the percentage of at least 700 cells analyzed per cell line. Three independent differentiations. n=3 biological replicates. **(C)** iPSC-derived MNs stained for Islet1 and Tuj1 after two weeks of maturation. Low magnification images of the immunocytochemical stainings revealed no major differences in the differentiation efficiency between the indicated iPSC-lines. The images are representative of at least three biological replicates. **(D)** Western blot showing the Plekhg5 expression in spinal cord lysates of wildtype and SOD1^{G93A} mice. **(E)** Quantification of the Plekhg5 expression normalized to Calnexin. n=3 biological replicates.

All data are shown as Mean ± SEM.

Supplementary Tables

Supplementary Table 1: Cell lines

iPSC Line	Genotype	Reference
34D6 - control iPSC-line #1	WT	[84]
IMR90- control iPSC line #2	WT	[77]
SOD1 R115G	heterozygous R115G	[85]
SOD1 D90A	homozygous D90A	[60]
SOD1 D90A igc - (isogenic control)	WT	[86]

Supplementary Table 2: Mouse strains

Name	Genetic background	Source
CD1	Crl:CD1(ICR)	Charles River 022CD1
Plekhg5$^{-/-}$	B6.Pekgh5/J	[17]
SOD1^{G93A}	B6SJL-TgN(SOD1-G93A)^{dl}1Gur/J	Jackson Lab, #002300
Thy1::YFP	B6.Cg-Tg(Thy1-YFPH)23Jrs/J	Jackson Lab, #003782
mRFP-GFP-LC3	C57BL/6-Tg(CAG-RFP/EGFP/Map1lc3b)1Hill/J	Jackson Lab, #027139

Supplementary Table 3: Plasmids

Name	Reference
pSIH-shLuciferase	[17]
pSIH-shPlekhg5#D	[17]
pSIH-shPlekhg5#E	[17]
pSIH-shPLEKHG5#1	this study
pSIH-shPLEKHG5#2	this study
pSIH-shPLEKHG5#3	this study
pSIH-shPLEKHG5#4	this study
pSIH-shAtg9#1	this study
pSIH-shAtg9#2	this study
pSIH-shAtg9#1	this study
pSIH-shStx17#1	this study
pSIH-shStx17#2	this study
pSIH-shSnap29#1	this study
pSIH-shSnap29#2	this study
pSIH-shSnap23#1	this study
pSIH-shSnap23#2	this study
FUV-3xFlag-LC3	this study

Supplementary Table 4: Primary antibodies for immunohistochemistry:

Name	Company	Catalogue number
Synaptophysin-1	Synaptic systems	101 400
Neurofilament heavy	Millipore Sigma	AB5539
Sod1 (mouse)	Enzo Life Science	ADI-SOD-100-F
SOD1 (human) (71G8)	Cell Signaling Technologies	4266
Lamp1	Invitrogen	14-1071-82
Lamp1	DSHB	1D4B
ChAT	Millipore Sigma	ab144P

GFP	Abcam	ab13970
RFP	Rockland	600-401-379
p62	Progen	GP62-C
CD68	Bio-Rad	MCA1957T
CathepsinD	Santa Cruz Biotechnology	6494
Iba1	Synaptic systems	234 004
Ubiquitin	Proteintech	10201-2-AP
Tau	Sigma-Aldrich	T6402
TDP43	Proteintech	22309-1-AP
Islet	Synaptic systems	406 003

Supplementary Table 5: Primary antibodies for Western blot:

Name	Company	Catalogue number
Sod1 (mouse)	Enzo Life Science	ADI-SOD-100-F
SOD1 (human) (71G8)	Cell Signaling Technologies	4266
Actin	Santa Cruz Biotechnology	Sc-8432
TUJ-1	Neuromics	MO15013
Atg9	Abcam	ab108338
LC3B	Novus Biologicals	NB100-2220
Atg5	Cell Signaling Technologies	12994
Calnexin	SicGen antibodies	AB0041-200
anti-GM130	BD Biosciences	610822
Lamp-1	Invitrogen	14-1071-82
Cytochrome C	Santa Cruz Biotechnology	sc-13156
Tsg101	Proteintech	14497-1-AP
Flag M2 Agarose	Millipore Sigma	A2220
Flag	Sigma life sciences	F7425-2MG
Cathepsin D	Davids Biotechnology	Custome made
Adaptin y	BD Biosciences	610385
elF2α	Cell Signaling Technologies	3597
Plekhg5	Proteintech	19830-1-AP
Rab5a	Cell Signaling Technologies	46449
Snap23	Synaptic Systems	111 205
Snap29	Synaptic Systems	111 303
Syntaxin17	Proteintech	17815-1-AP
Rab26	Synaptic Systems	269 011
Histone H3	Abcam	ab1791

Supplementary Table 6: Oligonucleotides:

Name	Sequence 5´- 3´	Company	Reference
Ccl3 qPCR Fwd	TCT CCT ACA GCC GGA AGA TTC	metabion	this study
Ccl3 qPCR Rev	CTT TGG AGT CAG CGC AGA TC	metabion	this study
Ccl4 qPCR Fwd	TCC CAC TTC CTG CTG TTT CT	metabion	this study
Ccl4 qPCR Rev	ATG TAC TCA GTG ACC CAG GG	metabion	this study
Ccl6 qPCR Fwd	TGC CAC ACA GAT CCC ATG TA	metabion	this study
Ccl6 qPCR Rev	CTC TGA ACT CTC CGA TCG CT	metabion	this study
Ccl9 qPCR Fwd	GCC TGT CCT ATA ACT CAC GGA	metabion	this study
Ccl9 qPCR Rev	TTG TAG GTC CGT GGT TGT GA	metabion	this study
CD68 qPCR Fwd	ACA AAA CCA AGG TCC AGG GA	metabion	this study

CD68 qPCR Rev	CAC ATT GTA TTC CAC CGC CA	metabion	this study
Trem2 qPCR Fwd	ATG CTG GAG ATC TCT GGG TC	metabion	this study
Trem2 qPCRRev	AGA AGA ATG GAG GTG GGT GG	metabion	this study

Name	Sequence 5´- 3´	Company	Reference
Plekhg5$^{-/-}$, Geno Fwd	TAAAAGCTGGCAGCCTGAAT	metabion	[17]
Plekhg5$^{-/-}$, Geno Genetrap-Rev	GCTAGCACAACCCCTCACTC	metabion	[17]
Plekhg5$^{-/-}$, Geno Rev	ACCCCAAGGTCTGTCCTCTT	metabion	[17]
Fwd SOD Ex 4f	CAT CAG CCC TAA TCC ATC TGA	metabion	Jackson Lab, oIMR0113
Rev SOD Ex 4r	CGC GAC TAA CAA TCA AAG TGA	metabion	Jackson Lab, oIMR0114
Thy1::YFP	TCC TTG AAG AAG ATG GTC CG	metabion	Jackson Lab, olMR1416
Thy1::YFP	AAG TTC ATC TGC ACC ACC	metabion	Jackson Lab, olMR0872
Atg5$^{fl/fl}$; 1	GAA TAT GAA GGC ACA CCC CTG AAA TG	metabion	[2]
Atg5$^{fl/fl}$; 2	ACA ACG TCG AGC ACA GCT GCG CAA GG	metabion	[2]
Atg5$^{fl/fl}$; 3	GTA CTG CAT AAT GGT TTA ACT CTT GC	metabion	[2]
mRFP-GFP-LC3 Transgene Fwd A GFP	CAT GGA CGA GCT GTA CAA GT	metabion	Jackson Lab, #24935
mRFP-GFP-LC3 Transgene Rev A Map1LC3B;	CAC CGT GAT CAG GTA CAA GGA	metabion	Jackson Lab, #24936
mRFP-GFP-LC3 Internal positive control Fwd A,	CTA GGC CAC AGA ATT GAA AGA TCT	metabion	Jackson Lab, oIMR7338
mRFP-GFP-LC3 Internal positive control Rev A,	GTA GGT GGA AAT TCT AGC ATC ATC C	metabion	Jackson Lab, oIMR7339

sh-RNA	Sequence 5´ - 3´	Company	Reference
sh-Atg9#1 sense	GATCCTGTAGGAGCAGGATGGAAATACTTCCTG TCAGATATTTCCATCCTGCTCCTACATTTTTG	metabion	this study
sh-Atg9#1 antisense	AATTCAAAAATGTAGGAGCAGGATGGAAATAT CTGACAGGAAGTATTTCCATCCTGCTCCTACA G	metabion	this study
sh-Atg9#2 sense	GATCCGTGGACTATGACATCCTATTTCTTCCTGT CAGAGTGGACTATGACATCCTATTTTTTTG	metabion	this study
sh-Atg9#2 antisense	AATTCAAAAAGTGGACTATGACATCCTATTTTC TGACAGGAAGGTGGACTATGACATCCTATTTG	metabion	this study

sh-PLEKHG5#1 sense	GATCCGAATGCTTCACTCTGAAATTTCTTCCTGTCAGAAAATTTCAGAGTGAAGCATTCTTTTTG	metabion	this study
sh-PLEKHG5#1 antisense	AATTCAAAAAGAATGCTTCACTCTGAAATTTTCTGACAGGAAGAAATTTCAGAGTGAAGCATTCG	metabion	this study
sh-PLEKHG5#2 sense	GATCCGAGGTGCTGCTGCCTGTATTTCTTCCTGTCAGAAAATACAGGCAGCAGCACCTCTTTTTG	metabion	this study
sh-PLEKHG5#2 antisense	AATTCAAAAAGAGGTGCTGCTGCCTGTATTTTCTGACAGGAAGAAATACAGGCAGCAGCACCTCG	metabion	this study
sh-PLEKHG5#3 sense	GATCCGACAAGCTCCTGAAGGAATTTCTTCCTGTCAGAAAATTCCTTCAGGAGCTTGTCTTTTTG	metabion	this study
sh-PLEKHG5#3 antisense	AATTCAAAAAGACAAGCTCCTGAAGGAATTTTCTGACAGGAAGAAATTCCTTCAGGAGCTTGTCG	metabion	this study
sh-PLEKHG5#4 sense	GATCCTGGCTGGGTGGACACCATTTACTTCCTGTCAGATAAATGGTGTCCACCCAGCCATTTTTG	metabion	this study
sh-PLEKHG5#4 antisense	AATTCAAAAATGGCTGGGTGGACACCATTTATCTGACAGGAAGTAAATGGTGTCCACCCAGCCAG	metabion	this study
sh-Stx17#1 sense	GATCCCAGAGTCTGACTCAGATATATCTTCCTGTCAGAATATATCTGAGTCAGACTCTGTTTTTG	metabion	this study
sh-Stx17#1 antisense	AATTCAAAAACAGAGTCTGACTCAGATATATTCTGACAGGAAGATATATCTGAGTCAGACTCTGG	metabion	this study
sh-Stx17#2 sense	GATCCACCTTAGAAGCGGACTTAATTCTTCCTGTCAGAAATTAAGTCCGCTTCTAAGGTTTTTG	metabion	this study
sh-Stx17#2 antisense	AATTCAAAAAACCTTAGAAGCGGACTTAATTTCTGACAGGAAGAATTAAGTCCGCTTCTAAGGTG	metabion	this study
sh-Snap29#1 sense	GATCCGAGTGTGTTTGGAGGATTTATCTTCCTGTCAGAATAAATCCTCCAAACACACTCTTTTTG	metabion	this study
sh-Snap29#1 antisense	AATTCAAAAAGAGTGTGTTTGGAGGATTTATTCTGACAGGAAGATAAATCCTCCAAACACACTCG	metabion	this study
sh-Snap29#2 sense	GATCCGACAAGCTAGATGTCAATATACTTCCTGTCAGATATATTGACATCTAGCTTGTCTTTTTG	metabion	this study
sh-Snap29#2 antisense	AATTCAAAAAGACAAGCTAGATGTCAATATATCTGACAGGAAGTATATTGACATCTAGCTTGTCG	metabion	this study
sh-Snap23#1 sense	GATCCAGAAGGCATGGACCAAATAAACTTCCTGTCAGATTTATTTGGTCCATGCCTTCTTTTTTG	metabion	this study
sh-Snap23#1 antisense	AATTCAAAAAAGAAGGCATGGACCAAATAAATCTGACAGGAAGTTTATTTGGTCCATGCCTTCTG	metabion	this study
sh-Snap23#2 sense	GATCCGCCAGTGGTGGATACATTAAACTTCCTGTCAGATTTAATGTATCCACCACTGGCTTTTTG	metabion	this study
sh-Snap23#2 antisense	AATTCAAAAAGCCAGTGGTGGATACATTAAATCTGACAGGAAGTTTAATGTATCCACCACTGGCG	metabion	this study
