# Supplementary material for: Plekhg5 controls the unconventional secretion of Sod1 by presynaptic secretory autophagy
Source: Nat Commun. 2024 Oct 4;15:8622. doi: 10.1038/s41467-024-52875-5 (PMC11452647; doi:10.1038/s41467-024-52875-5)
Supplement: Supplementary file 3 — Reporting Summary [file 41467_2024_52875_MOESM3_ESM.pdf]

nature portfolio

Corresponding author(s): Dr. Patrick Lüningschrör

Last updated by author(s): Aug 30, 2024

Reporting Summary

Nature Portfolio wishes to improve the reproducibility of the work that we publish. This form provides structure for consistency and transparency in reporting. For further information on Nature Portfolio policies, see our Editorial Policies and the Editorial Policy Checklist.

Statistics

For all statistical analyses, confirm that the following items are present in the figure legend, table legend, main text, or Methods section.

n/a	Confirmed	
☐	☒	The exact sample size (*n*) for each experimental group/condition, given as a discrete number and unit of measurement
☐	☒	A statement on whether measurements were taken from distinct samples or whether the same sample was measured repeatedly
☐	☒	The statistical test(s) used AND whether they are one- or two-sided<br>*Only common tests should be described solely by name; describe more complex techniques in the Methods section.*
☒	☐	A description of all covariates tested
☐	☒	A description of any assumptions or corrections, such as tests of normality and adjustment for multiple comparisons
☐	☒	A full description of the statistical parameters including central tendency (e.g. means) or other basic estimates (e.g. regression coefficient) AND variation (e.g. standard deviation) or associated estimates of uncertainty (e.g. confidence intervals)
☐	☒	For null hypothesis testing, the test statistic (e.g. F, t, r) with confidence intervals, effect sizes, degrees of freedom and P value noted<br>*Give P values as exact values whenever suitable.*
☒	☐	For Bayesian analysis, information on the choice of priors and Markov chain Monte Carlo settings
☒	☐	For hierarchical and complex designs, identification of the appropriate level for tests and full reporting of outcomes
☐	☒	Estimates of effect sizes (e.g. Cohen's *d*, Pearson's *r*), indicating how they were calculated

Our web collection on statistics for biologists contains articles on many of the points above.

Software and code

Policy information about availability of computer code

Data collection	No software was used for Data collection
Data analysis	Graphpd Prism (version 9), ImageJ (version 1.54f), Deepflash2 (https://github.com/matjesg/deepflash2, version 0.2.4), Imaris (version 7.7x), bcl2fastq2 (version 2.20.0.422), STAR (version 2.7.2b).

For manuscripts utilizing custom algorithms or software that are central to the research but not yet described in published literature, software must be made available to editors and reviewers. We strongly encourage code deposition in a community repository (e.g. GitHub). See the Nature Portfolio guidelines for submitting code & software for further information.

Data

Policy information about availability of data

All manuscripts must include a data availability statement. This statement should provide the following information, where applicable:

- Accession codes, unique identifiers, or web links for publicly available datasets
- A description of any restrictions on data availability
- For clinical datasets or third party data, please ensure that the statement adheres to our policy

Source data are provided within this paper. The raw RNA-eq data have been deposited to the Gene Expression Omnibus (GEO) with dataset identifier GSE269588. https://www.ncbi.nlm.nih.gov/geo/query/acc.cgi?acc=GSE269588.

Research involving human participants, their data, or biological material

Policy information about studies with human participants or human data. See also policy information about sex, gender (identity/presentation), and sexual orientation and race, ethnicity and racism.

Reporting on sex and gender	N/A
Reporting on race, ethnicity, or other socially relevant groupings	N/A
Population characteristics	N/A
Recruitment	N/A
Ethics oversight	N/A

Note that full information on the approval of the study protocol must also be provided in the manuscript.

Field-specific reporting

Please select the one below that is the best fit for your research. If you are not sure, read the appropriate sections before making your selection.

☒ Life sciences ☐ Behavioural & social sciences ☐ Ecological, evolutionary & environmental sciences

For a reference copy of the document with all sections, see nature.com/documents/nr-reporting-summary-flat.pdf

Life sciences study design

All studies must disclose on these points even when the disclosure is negative.

Sample size	Most experiments were carried out independently at least in triplicates. No calculations were done to predetermine sample size. Sample size was chosen based on authors experience and established standards for spinal cord tissue, cell lines, primary moto neurons and IPSCs from animal models.
Data exclusions	No data were excluded in this study.
Replication	To ensure reproducability, all experiments were carried out at least in triplicates. Replication was successful in all experiments.
Randomization	All experiments were carried out with primary motoneurons, tissue from mice with defined genetic background, NSC34 and IPSCs. For all experiments, treatment conditions and control with were selected from the same batch.
Blinding	Samples for cell culture with different experimental conditions were preocessed simultaniously such that blinding was not necessary. Spinal cord tissue samples from mice for quantification were blinded to ensure equal fluoresence analysis.

Reporting for specific materials, systems and methods

We require information from authors about some types of materials, experimental systems and methods used in many studies. Here, indicate whether each material, system or method listed is relevant to your study. If you are not sure if a list item applies to your research, read the appropriate section before selecting a response.

Materials & experimental systems

n/a	Involved in the study	
☐	☒	Antibodies
☐	☒	Eukaryotic cell lines
☒	☐	Palaeontology and archaeology
☐	☒	Animals and other organisms
☒	☐	Clinical data
☒	☐	Dual use research of concern
☒	☐	Plants

Methods

n/a	Involved in the study	
☒	☐	ChIP-seq
☒	☐	Flow cytometry
☒	☐	MRI-based neuroimaging

Antibodies

Antibodies used

Primary antibodies:
1. Guinea Pig Polyclonal anti-Synaptophysin1, Synaptic Systems, Cat# 101 004, RRID:AB_1210382, WB (1:8000), IF (1:500).
2. Chicken Polyclonal anti-Neurofilament heavy, Millipore, Cat# AB5539, RRID:AB_11212161, IF (1:500).
3. Rabbit Polyclonal anit-SOD1, Enzo Life Sciences, Cat# ADI-SOD-100, RRID:AB_10616253, WB (1:5000), IF (1:800).
4. Mouse Monoclonal anti-human SOD1, Cell Signaling Technology, Cat# 4266, RRID:AB_2193898, WB (1:4000).
5. Rat Monoclonal anti-Lamp1, Thermo Fisher Scientific, Cat# 14-1071-82, RRID:AB_657531, WB (1:1000), IF (1:500).
6. Mouse Monoclonal anti-Lamp1, DSHB, Cat# 1d4b, RRID:AB_2134500, WB (1:1000).
7. Goat Polyclonal anti-ChAT, Millipore, Cat# AB144P, RRID:AB_2079751, IF (1:600).
8. Chicken Polyclonal anti-GFP, Abcam, Cat# ab13970, RRID:AB_300798, WB (1:500), IF (1:500).
9. Rabbit Polyclonal anti-RFP, Rockland, Cat# 600-401-379, RRID:AB_2209751, WB (1:4000), IF (1:500).
10. Guinea Pig Polyclonal anti-p62, Progen, Cat# GP62-C, RRID:AB_2687531, WB (1:2000), IF (1:500).
11. Rat Monoclonal anti-CD68, Bio-Rad, Cat# MCA1957T, RRID:AB_2074849, IF (1:800).
12. Goat Polyclonal anti-CathD, Santa Cruz Biotechnology, Cat# sc-6494, RRID:AB_2087097, WB (1:1000), IF (1:600).
13. Guinea Pig Polyclonal anti-Iba1, Synaptic Systems, Cat# 234 004, RRID:AB_2493179, IF (1:800).
14. Rabbit Polyclonal anti-ubiquitin, Agilent, Cat# Z0458, RRID:AB_2315524, WB (1:20000), IF (1:500).
15. Mouse Monoclonal anti-actin, Santa Cruz Biotechnology, Cat# sc-8432, RRID:AB_626630, WB (1:5000).
16. Mouse Monoclonal anti-TUJ1, Neuromics, Cat# MO15013, RRID:AB_2737114, WB (1:10000), IF (1:500).
17. Rabbit Monoclonal anti-Atg9a, Abcam, Cat# ab108338, RRID:AB_10863880, WB (1:4000), IF (1:400).
18. Rabbit Polyclonal anti-LC3B, Novus, Cat# NB100-2220, RRID:AB_10003146, WB (1:10000).
19. Rabbit Monoclonal anti-Atg5, Cell Signaling Technology, Cat# 12994, RRID:AB_2630393, WB (1:4000).
20. Goat Polyclonal anti-Calnexin, SICGEN, Cat# AB0041, RRID:AB_2333116, WB (1:10000).
21. Mouse Monoclonal anti-GM130, BD Biosciences, Cat# 610822, RRID:AB_398141, WB (1:2000).
22. Mouse Monoclonal anti-Cytochrome-C, Santa Cruz Biotechnology, Cat# sc-13156, RRID:AB_627385, WB (1:1000).
23. Rabbit Polyclonal anti-Tsg101, Proteintech, Cat# 14497-1-AP, RRID:AB_2208090, WB (1:1000).
24. Rabbit Polyclonal anti-Flag, Sigma-Aldrich, Cat# F7425, RRID:AB_439687, WB (1:10000).
25. Mouse Monoclonal anti-Adaptin γ, BD Biosciences, Cat# 610385, RRID:AB_397768, WB (1:1000).
26. Rabbit Monoclonal anti-elf2a, Cell Signaling Technology, Cat# 3597, RRID:AB_390740, WB (1:4000).
27. Rabbit Polyclonal anti-Plekhg5, Proteintech, Cat# 19830-1-AP, RRID:AB_10858324, WB (1:2000).
28. Mouse Monoclonal anti-Rab5a, Cell Signaling Technology, Cat# 46449, RRID:AB_2799303, WB (1:5000).
29. Guinea Pig Polyclonal anti-Snap23, Synaptic Systems, Cat# 111 205, RRID:AB_10697033, WB (1:500).
30. Rabbit Polyclonal anti-Snap29, Synaptic Systems, Cat# 111 303, RRID:AB_2302217, WB (1:1000).
31. Rabbit Polyclonal anti-Syntaxin17, Proteintech, Cat# 17815-1-AP, RRID:AB_2255542, WB (1:2000).
32. Mouse Monoclonal anti-Rab26, Synaptic Systems, Cat# 269 011, RRID:AB_2619993, WB (1:1000).
33. Rabbit Polyclonal anti-Histon3, Abcam, Cat# ab1791, RRID:AB_302613, WB (1:5000).
34. Rabbit anti-CathedpsinD, Davids Biotechnology, Custume made, RRID: not available, WB (1:2000).
35. Rabbit Polyclonal anti-Tau, Sigma-Aldrich, Cat# T6402, RRID:AB_261728, IF (1:40).
36. Rabbit Polyclonal anti-TDP43, Proteintech, Cat# 22309-1-AP, RRID:AB_11182943, IF (1:400).
37. Rabbit Polyclonal anti-Islet1, Synaptic Systems, Cat# 406 003, RRID:AB_2725764, IF (1:500).

Secondary antibodies:
1. Donkey Polyclonal anti-Chicken (Alexa Fluor® 488, Jackson ImmunoResearch Labs, Cat# 703-545-155, RRID:AB_2340375, IF (1:800).
2. Donkey Polyclonal anti-Rabbit (Cy™3 Conjugated), Jackson ImmunoResearch Labs, Cat# 711-165-152, RRID:AB_2307443, IF (1:800).
3. Donkey Polyclonal anti-Rabbit (Cy™5 Conjugated), Jackson ImmunoResearch Labs, Cat# 711-175-152, RRID:AB_2340607, IF (1:800).
4. Donkey Polyclonal anti-Rat (Alexa Fluor® 488), Jackson ImmunoResearch Labs, Cat# 712-545-150, RRID:AB_2340683, IF (1:800).
5. Donkey Polyclonal anti-Rat (Cy™3 Conjugated), Jackson ImmunoResearch Labs, Cat# 712-165-153, RRID:AB_2340667, IF (1:800).
6. Donkey Polyclonal anti-Guinea Pig (Cy™5 Conjugated), Jackson ImmunoResearch Labs, Cat# 706-175-148, RRID:AB_2340462, IF (1:800).
7. Donkey Polyclonal anti-Goat (Cy™5 Conjugated), Jackson ImmunoResearch Labs, Cat# 705-175-147, RRID:AB_2340415, IF (1:800).
8. Donkey Polyclonal anti-Mouse (Cy™3 Conjugated), Jackson ImmunoResearch Labs, Cat# 715-165-150, RRID:AB_2340813, IF (1:800).
9. Donkey Polyclonal anti-Goat (CF®405S Conjugated), Biotium, Cat# 20416-500uL, RRID:not available, IF (1:800).
10. Horse Polyclonal anti-Mouse (Peroxidase Conjugated), Cell Signaling Technology, Cat# 7076, RRID:AB_330924, WB (1:10000).
11. Donkey Polyclonal anti-Rabbit (Peroxidase Conjugated), Jackson ImmunoResearch Labs, Cat# 711-035-152, RRID:AB_10015282, WB (1:10000).
12. Donkey Polyclonal anti-Chicken (Peroxidase Conjugated), Jackson ImmunoResearch, Labs Cat# 703-035-155, RRID:AB_10015283, WB (1:10000).
13. Donkey Polyclonal anti-Guinea Pig (Peroxidase Conjugated), Jackson ImmunoResearch Labs, Cat# 706-035-148, RRID:AB_2340447, WB (1:10000).
14. Donkey Polyclonal anti-Rat (Peroxidase Conjugated), Jackson ImmunoResearch Labs, Cat# 712-035-153, RRID:AB_2340639, WB (1:10000).
15. Donkey Polyclonal anti- Goat (Peroxidase Conjugated), Millipore, Cat# AP180P, RRID:AB_92573, WB (1:10000).

Validation

Primary antibodies:
1. Guinea Pig Polyclonal anti-Synaptophysin1, Synaptic Systems, Cat# 101 004, PRID:AB_1210382, WB (1:8000), IF (1:500). Validation data provided by the supplier (https://www.sysy.com/product/101004).
2. Chicken Polyclonal anti-Neurofilament heavy, Millipore, Cat# AB5539, RRID:AB_11212161, IF (1:500). Validation data provided by the supplier (https://www.merckmillipore.com/DE/de/product/Anti-Neurofilament-H-Antibody,MM_NF-AB5539#anchor_Product%20Information).
3. Rabbit Polyclonal anit-SOD1, Enzo Life Sciences ,Cat# ADI-SOD-100, RRID:AB_10616253), WB (1:5000), IF (1:800). Validation data provided by the supplier (https://www.enzo.com/product/cu-zn-sod-polyclonal-antibody-2/).

4. Mouse Monoclonal anti-human SOD1, Cell Signaling Technology, Cat# 4266, RRID:AB_2193898, WB (1:4000). Validation data provided by the supplier (https://www.cellsignal.com/products/primary-antibodies/sod1-71g8-mouse-mab/4266).
5. Rat Monoclonal anti-Lamp1, Thermo Fisher Scientific, Cat# 14-1071-82, RRID:AB_657531, WB (1:1000), IF (1:500). Validation data provided by the supplier (https://www.thermofisher.com/antibody/product/CD107a-LAMP-1-Antibody-clone-eBio1D4B-1D4B-Monoclonal/14-1071-82).
6. Mouse Monoclonal anti-Lamp1, DSHB, Cat# 1d4b, RRID:AB_2134500, WB (1:1000). Validation data provided by the supplier (https://dshb.biology.uiowa.edu/1D4B).
7. Goat Polyclonal anti-ChAT, Millipore, Cat# AB144P, RRID:AB_2079751, IF (1:600). Validation data provided by the supplier (https://www.merckmillipore.com/DE/de/product/Anti-Choline-Acetyltransferase-Antibody,MM_NF-AB144P).
8. Chicken Polyclonal anti-GFP, Abcam, Cat# ab13970, RRID:AB_300798, WB (1:500), IF (1:500). Validation data provided by the supplier (https://www.abcam.com/en-de/products/primary-antibodies/gfp-antibody-ab13970).
9. Rabbit Polyclonal anti-RFP, Rockland, Cat# 600-401-379, RRID:AB_2209751, WB (1:4000), IF (1:500). Validation data provided by the supplier (https://www.rockland.com/categories/primary-antibodies/rfp-antibody-pre-adsorbed-600-401-379/).
10. Guinea Pig Polyclonal anti-p62, Progen, Cat# GP62-C, RRID:AB_2687531, WB (1:2000), IF (1:500). Validation data provided by the supplier (https://www.progen.com/anti-p62-SQSTM1-C-terminus-guinea-pig-polyclonal-serum/GP62-C).
11. Rat Monoclonal anti-CD68, Bio-Rad, Cat# MCA1957T, RRID:AB_2074849, IF (1:800). Validation data provided by the supplier (https://www.bio-rad-antibodies.com/monoclonal/mouse-cd68-antibody-fa-11-mca1957.html?f=purified).
12. Goat Polyclonal anti-CathD, Santa Cruz Biotechnology, Cat# sc-6494, RRID:AB_2087097, WB (1:1000), IF (1:600). Discontinued, no validation available (https://www.citeab.com/antibodies/808945-sc-6494-cathepsin-d-antibody-g-19).
13. Guinea Pig Polyclonal anti-Iba1, Synaptic Systems, Cat# 234 004, RRID:AB_2493179, IF (1:800). Discontinued, no validation available (https://www.citeab.com/antibodies/2042911-234-004-iba1).
14. Rabbit Polyclonal anti-ubiquitin, Agilent, Cat# Z0458, RRID:AB_2315524, WB (1:20000), IF (1:500). Discontinued, no validation available (https://www.citeab.com/antibodies/3382935-z0458-ubiquitin).
15. Mouse Monoclonal anti-actin, Santa Cruz Biotechnology, Cat# sc-8432, RRID:AB_626630, WB (1:5000). Validation data provided by the supplier (https://www.scbt.com/de/p/actin-antibody-c-2).
16. Mouse Monoclonal anti-TUJ1, Neuromics, Cat# MO15013, RRID:AB_2737114, WB (1:10000), IF (1:500). Validation data provided by the supplier (https://www.neuromics.com/ittrium/reference/D8x1b63x8x1).
17. Rabbit Monoclonal anti-Atg9a, Abcam, Cat# ab108338, RRID:AB_10863880, WB (1:4000), IF (1:400). Validation in our lab by sh-Atg9 knock down.
18. Rabbit Polyclonal anti-LC3B, Novus, Cat# NB100-2220, RRID:AB_10003146, WB (1:10000). Validation data provided by the supplier (https://www.novusbio.com/products/lc3b-antibody_nb100-2220).
19. Rabbit Monoclonal anti-Atg5, Cell Signaling Technology, Cat# 12994, RRID:AB_2630393, WB (1:4000). Validation in our lab by Cre flox/flox Atg5 knock out.
20. Goat Polyclonal anti-Calnexin, SICGEN, Cat# AB0041, RRID:AB_2333116, WB (1:10000). Validation data provided by the supplier (https://www.origene.com/catalog/antibodies/primary-antibodies/ab0041-200/calnexin-canx-goat-polyclonal-antibody).
21. Mouse Monoclonal anti-GM130, BD Biosciences, Cat# 610822, RRID:AB_398141, WB (1:2000). Validation data provided by the supplier (https://www.bdbiosciences.com/en-us/products/reagents/microscopy-imaging-reagents/immunofluorescence-reagents/purified-mouse-anti-gm130.610822).
22. Mouse Monoclonal anti-Cytochrome-C, Santa Cruz Biotechnology, Cat# sc-13156, RRID:AB_627385, WB (1:1000). Validation data provided by the supplier (https://www.scbt.com/de/p/cytochrome-c-antibody-a-8).
23. Rabbit Polyclonal anti-Tsg101, Proteintech, Cat# 14497-1-AP, RRID:AB_2208090, WB (1:1000). Validation data provided by the supplier (https://www.ptglab.com/de/products/TSG101-Antibody-14497-1-AP.htm).
24. Rabbit Polyclonal anti-Flag, Sigma-Aldrich, Cat# F7425, RRID:AB_439687, WB (1:10000). Validation data provided by the supplier (https://www.sigmaaldrich.com/DE/de/product/sigma/f7425).
25. Mouse Monoclonal anti-Adaptin γ, BD Biosciences, Cat# 610385, RRID:AB_397768, WB (1:1000). Validation data provided by the supplier (https://www.bdbiosciences.com/en-ca/products/reagents/microscopy-imaging-reagents/immunofluorescence-reagents/purified-mouse-anti-adaptin.610385).
26. Rabbit Monoclonal anti-elf2a, Cell Signaling Technology, Cat# 3597, RRID:AB_390740, WB (1:4000). Validation data provided by the supplier (https://www.cellsignal.com/products/primary-antibodies/phospho-eif2a-ser51-119a11-rabbit-mab/3597).
27. Rabbit Polyclonal anti-Plekhg5, Proteintech, Cat# 19830-1-AP, RRID:AB_10858324, WB (1:2000). Validation in our lab by sh-Plekhg5 knock down.
28. Mouse Monoclonal anti-Rab5a, Cell Signaling Technology, Cat# 46449, RRID:AB_2799303, WB (1:5000). Validation data provided by the supplier (https://www.cellsignal.com/products/primary-antibodies/rab5a-e6n8s-mouse-mab/46449).
29. Guinea Pig Polyclonal anti-Snap23, Synaptic Systems, Cat# 111 205, RRID:AB_10697033, WB (1:500). Validation in our lab by sh-Snap23 knock down.
30. Rabbit Polyclonal anti-Snap29, Synaptic Systems, Cat# 111 303, RRID:AB_2302217, WB (1:1000). Validation in our lab by sh-Snap29 knock down.
31. Rabbit Polyclonal anti-Syntaxin17, Proteintech, Cat# 17815-1-AP, RRID:AB_2255542, WB (1:2000). Validation in our lab by sh-Syntaxin17 knock down.
32. Mouse Monoclonal anti-Rab26, Synaptic Systems, Cat# 269 011, RRID:AB_2619993, WB (1:1000). Validation in our lab by sh-Rab26 knock down.
33. Rabbit Polyclonal anti-Histon3, Abcam, Cat# ab1791, RRID:AB_302613, WB (1:5000). Validation data provided by the supplier (Validation data provided by the supplier).
34. Rabbit anti-CathedpsinD, Davids Biotechnology, Custume made, RRID: not available, WB (1:2000). Validated in our lab by omission of primary antibody in immunohistochemistry.
35. Rabbit Polyclonal anti-Tau, Sigma-Aldrich, Cat# T6402, RRID:AB_261728, IF (1:40). Validation data provided by the supplier (Validation data provided by the supplier).
36. Rabbit Polyclonal anti-TDP43, Proteintech, Cat# 22309-1-AP, RRID:AB_11182943, IF (1:400). Validation data provided by the supplier (Validation data provided by the supplier).
37. Rabbit Polyclonal anti-Islet1, Synaptic Systems, Cat# 406 003, RRID:AB_2725764, IF (1:500). Validation data provided by the supplier (Validation data provided by the supplier).

Secondary antibodies:
1. Donkey Polyclonal anti-Chicken (Alexa Fluor® 488, Jackson ImmunoResearch Labs, Cat# 703-545-155, RRID:AB_2340375, IF (1:800). Validated in our lab by omission of primary antibody, absence of target antigen and used under similar conditions for other projects.

2. Donkey Polyclonal anti-Rabbit (Cy™3 Conjugated), Jackson ImmunoResearch Labs, Cat# 711-165-152, RRID:AB_2307443, IF (1:800). Validated in our lab by omission of primary antibody, absence of target antigen and used under similar conditions for other projects.
3. Donkey Polyclonal anti-Rabbit (Cy™5 Conjugated), Jackson ImmunoResearch Labs, Cat# 711-175-152, RRID:AB_2340607, IF (1:800). Validated in our lab by omission of primary antibody, absence of target antigen and used under similar conditions for other projects.
4. Donkey Polyclonal anti-Rat (Alexa Fluor® 488), Jackson ImmunoResearch Labs, Cat# 712-545-150, RRID:AB_2340683.Validated in our lab by omission of primary antibody, absence of target antigen and used under similar conditions for other projects.
5. Donkey Polyclonal anti-Rat (Cy™3 Conjugated), Jackson ImmunoResearch Labs, Cat# 712-165-153, RRID:AB_2340667, IF (1:800). Validated in our lab by omission of primary antibody, absence of target antigen and used under similar conditions for other projects.
6. Donkey Polyclonal anti-Guinea Pig (Cy™5 Conjugated), Jackson ImmunoResearch Labs, Cat# 706-175-148, RRID:AB_2340462, IF (1:800). Validated in our lab by omission of primary antibody, absence of target antigen and used under similar conditions for other projects.
7. Donkey Polyclonal anti-Goat (Cy™5 Conjugated), Jackson ImmunoResearch Labs, Cat# 705-175-147, RRID:AB_2340415, IF (1:800). Validated in our lab by omission of primary antibody, absence of target antigen and used under similar conditions for other projects.
8. Donkey Polyclonal anti-mouse (Cy™3 Conjugated), Jackson ImmunoResearch Labs, Cat# 715-165-150, RRID:AB_2340813, IF (1:800). Validated in our lab by omission of primary antibody, absence of target antigen and used under similar conditions for other projects.
9. Donkey Polyclonal anti-Goat (CF®405S Conjugated), Biotium, Cat# 20416-500uL, RRID:not available, IF (1:800). Validated in our lab by omission of primary antibody, absence of target antigen and used under similar conditions for other projects.
10. Horse Polyclonal anti-Mouse (Peroxidase Conjugated), Cell Signaling Technology, Cat# 7076, RRID:AB_330924, WB (1:10000). Validated in our lab by omission of primary antibody, absence of target antigen and used under similar conditions for other projects.
11. Donkey Polyclonal anti-Rabbit (Peroxidase Conjugated), Jackson ImmunoResearch Labs, Cat# 711-035-152, RRID:AB_10015282, WB (1:10000). Validated in our lab by omission of primary antibody, absence of target antigen and used under similar conditions for other projects.
12. Donkey Polyclonal anti-Chicken (Peroxidase Conjugated), Jackson ImmunoResearch, Labs Cat# 703-035-155, RRID:AB_10015283, WB (1:10000). Validated in our lab by omission of primary antibody, absence of target antigen and used under similar conditions for other projects.
13. Donkey Polyclonal anti-Guinea Pig (Peroxidase Conjugated), Jackson ImmunoResearch Labs, Cat# 706-035-148, RRID:AB_2340447, WB (1:10000). Validated in our lab by omission of primary antibody, absence of target antigen and used under similar conditions for other projects.
14. Donkey Polyclonal anti-Rat (Peroxidase Conjugated), Jackson ImmunoResearch Labs, Cat# 712-035-153, RRID:AB_2340639, WB (1:10000). Validated in our lab by omission of primary antibody, absence of target antigen and used under similar conditions for other projects.
15. Donkey Polyclonal anti- Goat (Peroxidase Conjugated), Millipore, Cat# AP180P, RRID:AB_92573, WB (1:10000). Validated in our lab by omission of primary antibody, absence of target antigen and used under similar conditions for other projects.

Eukaryotic cell lines

Policy information about cell lines and Sex and Gender in Research

Cell line source(s)	NSC-34 cells (Cedarlane, cat. no. CLU140) and HEK293TN cells (System Biosiences, cat. no. LV900A-1)
Authentication	NSC-34 cells and HEK293TN cells were obtained commercially and were not authenticated.
Mycoplasma contamination	NSC-34 cells and HEK293TN cells tested negative for mycoplasma contamination.
Commonly misidentified lines (See ICLAC register)	No commonly misidentified cell lines were used in the study.

Animals and other research organisms

Policy information about studies involving animals; ARRIVE guidelines recommended for reporting animal research, and Sex and Gender in Research

Laboratory animals	CD1, Plekgh5-/-, SODG93A, Thy1::YFP and mRFP-GFP-LC3mice of both genders were housed in the animal facility of the Institute of Clinical Neurobiology at the University Hospital of Wuerzburg. Mice were maintained on a 12h/12h day/night cycle under conditions at 20-22°C and 55-65% humidity with food and water in abundant supply. Food was provided as hybrid pallets from Altromin (1318) during breeding and (1324) during housing and experiments. Breeding animals were between 6 and 20 weeks of age. Pregnancy in female mice was detected by daily plug control, and mouse embryos were isolated at E13 for generation of culture of primary motoneurons. Tissues were obtained from adult mice at defined ages as indicated in the manuscript.<br><br>CD1; Crl:CD1(ICR); Charles River 022CD1<br>Plekhg5-/-; B6.Pekgh5/J; DOI: 10.1038/s41467-017-00689-z<br>SOD1G93A; B6SJL-TgN(SOD1-G93A)dl1Gur/J; Jackson Lab, #002300<br>Thy1::YFP; B6.Cg-Tg(Thy1-YFPH)23Jrs/J; Jackson Lab, #003782<br>mRFP-GFP-LC3; C57BL/6-Tg(CAG-RFP/EGFP/Map1lc3b)1Hill/J; Jackson Lab, #027139
Wild animals	This study did not involve wild animals
Reporting on sex	Sex was not considered regarding embryonic mice used for motoneuron cultures. Animals for tissue harvesting were not discriminated between sex and iqually used.

Field-collected samples	This study did not involve samples collected from the field
Ethics oversight	All animal experiments were preformed strictly according to the regulations on animal protection of the German federal law and the Association of Assessment and Accreditation of Laboratory animal care, in agreement with and under the control of the local veterinary authority.

Note that full information on the approval of the study protocol must also be provided in the manuscript.

Plants

Seed stocks	*Report on the source of all seed stocks or other plant material used. If applicable, state the seed stock centre and catalogue number. If plant specimens were collected from the field, describe the collection location, date and sampling procedures.*
Novel plant genotypes	*Describe the methods by which all novel plant genotypes were produced. This includes those generated by transgenic approaches, gene editing, chemical/radiation-based mutagenesis and hybridization. For transgenic lines, describe the transformation method, the number of independent lines analyzed and the generation upon which experiments were performed. For gene-edited lines, describe the editor used, the endogenous sequence targeted for editing, the targeting guide RNA sequence (if applicable) and how the editor was applied.*
Authentication	*Describe any authentication procedures for each seed stock used or novel genotype generated. Describe any experiments used to assess the effect of a mutation and, where applicable, how potential secondary effects (e.g. second site T-DNA insertions, mosiacism, off-target gene editing) were examined.*
