# Supplementary figures and images for: Plekhg5 controls the unconventional secretion of Sod1 by presynaptic secretory autophagy (part 2 of 2)
Source: Nat Commun. 2024 Oct 4;15:8622. doi: 10.1038/s41467-024-52875-5 (PMC11452647; doi:10.1038/s41467-024-52875-5)

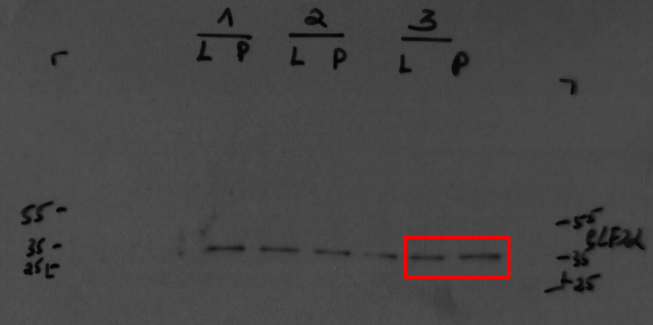

Supplement: Supplementary file 4 — Source Data [file 41467_2024_52875_MOESM4_ESM.zip › WB Full Scans/Figure_4/A/Fig.4_A_Cytosol_elF2a.tif]

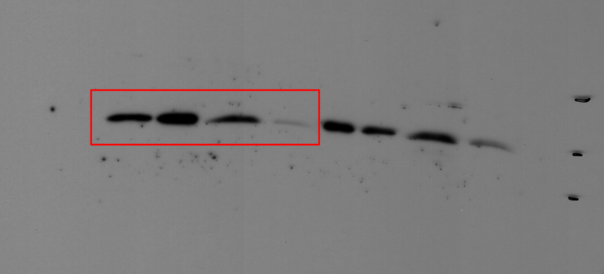

Supplement: Supplementary file 4 — Source Data [file 41467_2024_52875_MOESM4_ESM.zip › WB Full Scans/Figure_4/O/Fig.4_O_Sod1.tif]

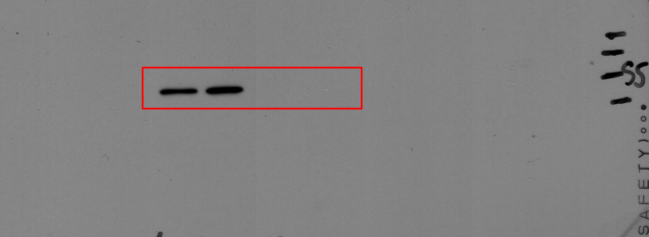

Supplement: Supplementary file 4 — Source Data [file 41467_2024_52875_MOESM4_ESM.zip › WB Full Scans/Figure_4/O/Fig.4_O_Tuj1.tif]

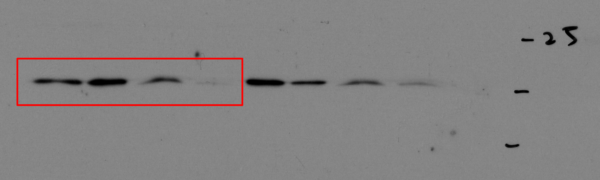

Supplement: Supplementary file 4 — Source Data [file 41467_2024_52875_MOESM4_ESM.zip › WB Full Scans/Figure_4/M/Fig.4_M_Sod1.tif]

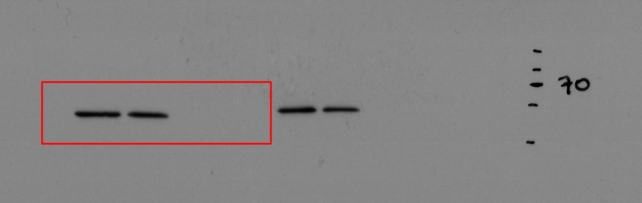

Supplement: Supplementary file 4 — Source Data [file 41467_2024_52875_MOESM4_ESM.zip › WB Full Scans/Figure_4/M/Fig.4_M_Tuj1.tif]

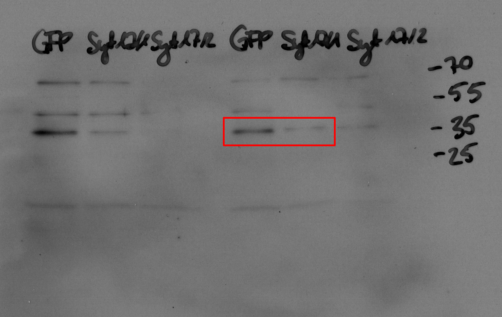

Supplement: Supplementary file 4 — Source Data [file 41467_2024_52875_MOESM4_ESM.zip › WB Full Scans/Figure_4/J/Fig.4_J_Stx17.tif]

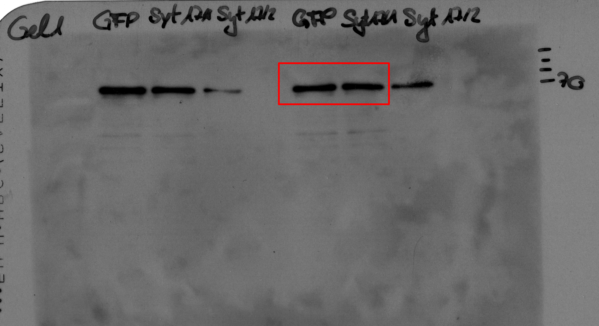

Supplement: Supplementary file 4 — Source Data [file 41467_2024_52875_MOESM4_ESM.zip › WB Full Scans/Figure_4/J/Fig.4_J_Calnexin.tif]

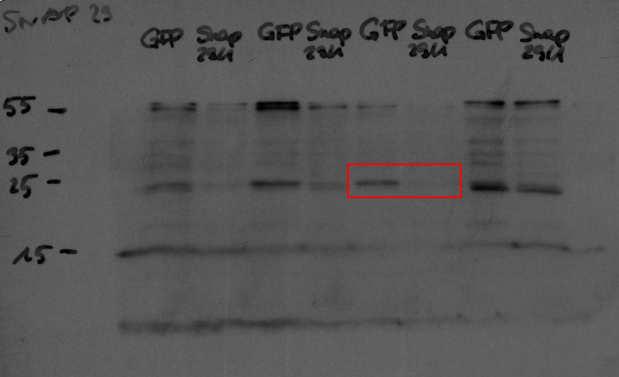

Supplement: Supplementary file 4 — Source Data [file 41467_2024_52875_MOESM4_ESM.zip › WB Full Scans/Figure_4/K/Fig.4_K_Snap29.tif]

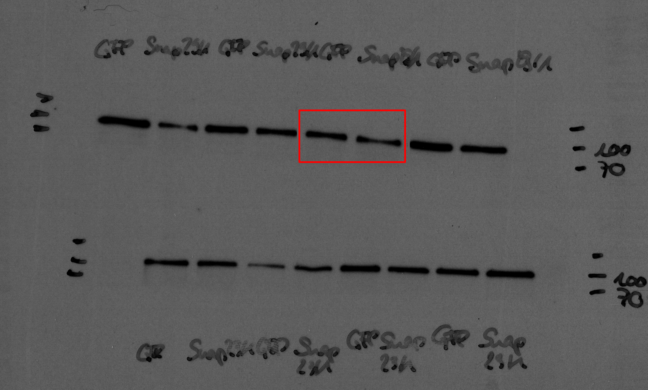

Supplement: Supplementary file 4 — Source Data [file 41467_2024_52875_MOESM4_ESM.zip › WB Full Scans/Figure_4/K/Fig.4_K_Calnexin.tif]

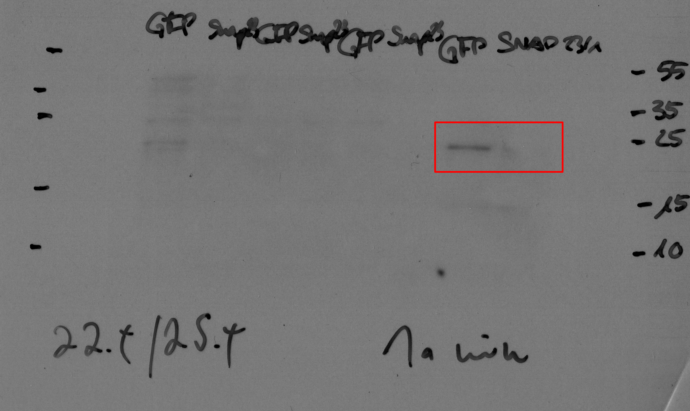

Supplement: Supplementary file 4 — Source Data [file 41467_2024_52875_MOESM4_ESM.zip › WB Full Scans/Figure_4/L/Fig.4_L_Snap23.tif]

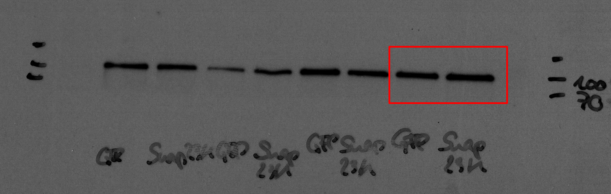

Supplement: Supplementary file 4 — Source Data [file 41467_2024_52875_MOESM4_ESM.zip › WB Full Scans/Figure_4/L/Fig.4_L_Calnexin.tif]
