# Supplementary figures and images for: Plekhg5 controls the unconventional secretion of Sod1 by presynaptic secretory autophagy (part 1 of 2)
Source: Nat Commun. 2024 Oct 4;15:8622. doi: 10.1038/s41467-024-52875-5 (PMC11452647; doi:10.1038/s41467-024-52875-5)

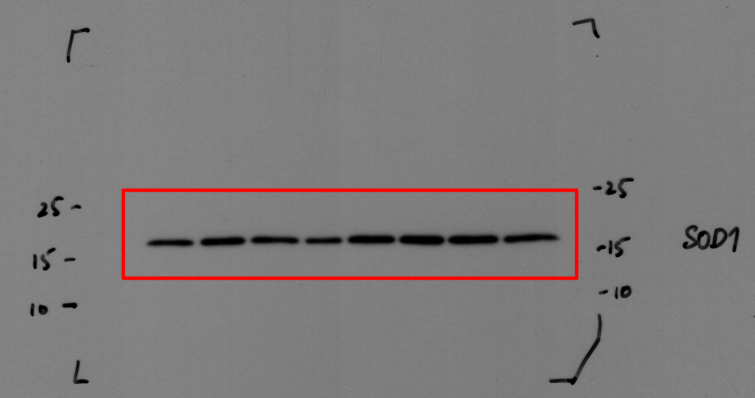

Supplement: Supplementary file 4 — Source Data [file 41467_2024_52875_MOESM4_ESM.zip › WB Full Scans/Figure_1/H/Fig.1_H-TX-100_soluble_Sod1.tif]

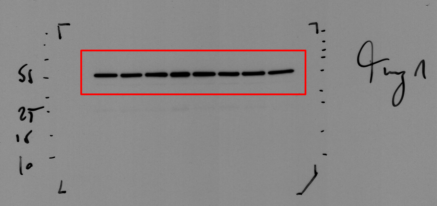

Supplement: Supplementary file 4 — Source Data [file 41467_2024_52875_MOESM4_ESM.zip › WB Full Scans/Figure_1/H/Fig.1_H-TX-100_soluble_Tuj1.tif]

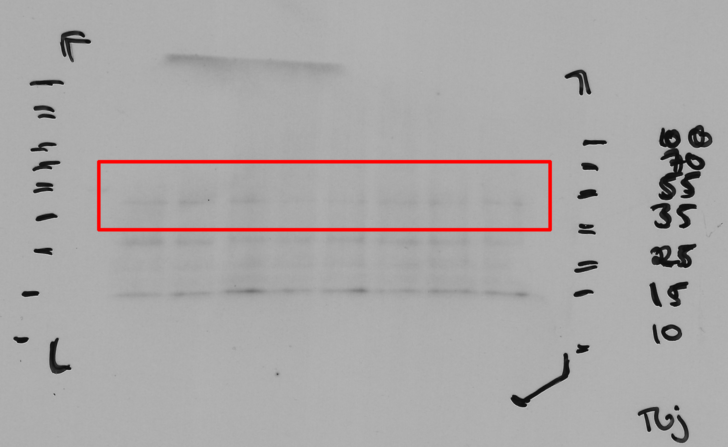

Supplement: Supplementary file 4 — Source Data [file 41467_2024_52875_MOESM4_ESM.zip › WB Full Scans/Figure_1/H/Fig.1_H-SDS-soluble_Tuj1.tif]

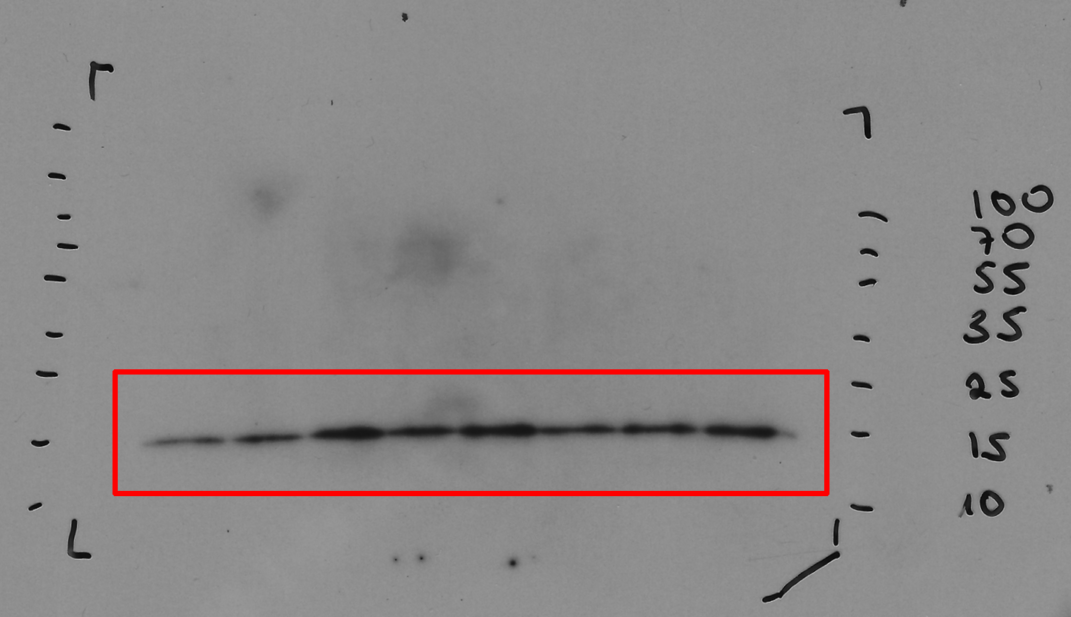

Supplement: Supplementary file 4 — Source Data [file 41467_2024_52875_MOESM4_ESM.zip › WB Full Scans/Figure_1/H/Fig.1_H-SDS-soluble_Sod1.tif]

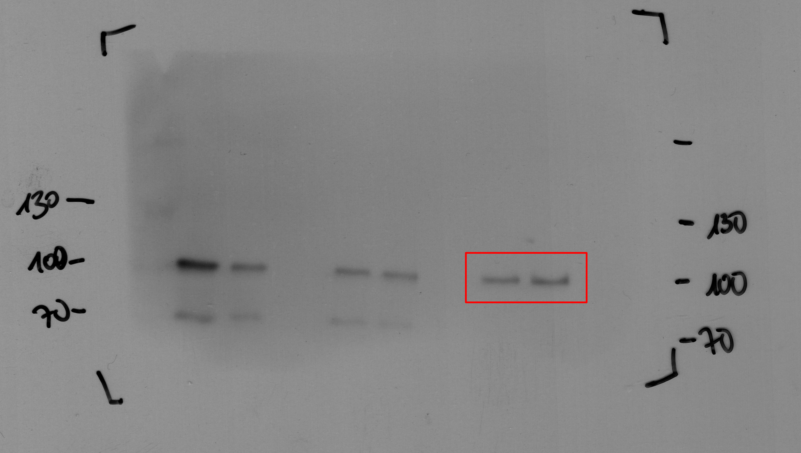

Supplement: Supplementary file 4 — Source Data [file 41467_2024_52875_MOESM4_ESM.zip › WB Full Scans/Figure_7/I/Fig.7_I_D90A-Calnexin.tif]

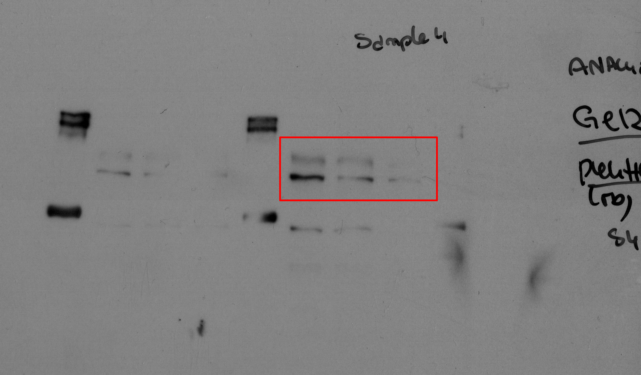

Supplement: Supplementary file 4 — Source Data [file 41467_2024_52875_MOESM4_ESM.zip › WB Full Scans/Figure_7/I/Fig.7_I_R115G-Plekhg5-LE.tif]

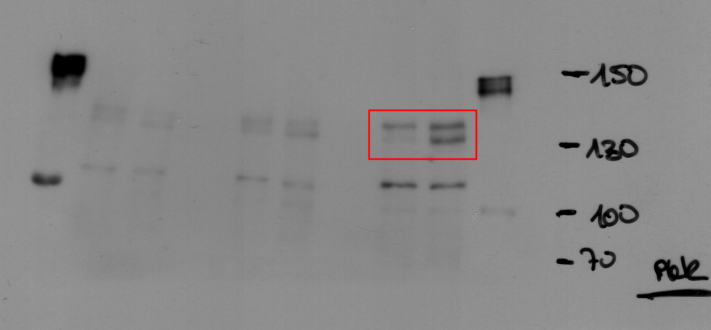

Supplement: Supplementary file 4 — Source Data [file 41467_2024_52875_MOESM4_ESM.zip › WB Full Scans/Figure_7/I/Fig.7_I_D90A-Plekhg5-LE.tif]

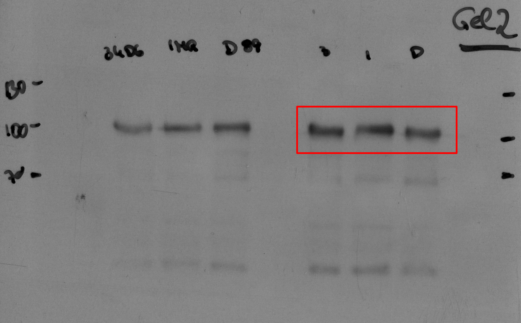

Supplement: Supplementary file 4 — Source Data [file 41467_2024_52875_MOESM4_ESM.zip › WB Full Scans/Figure_7/I/Fig.7_I_R115G-Calnexin.tif]

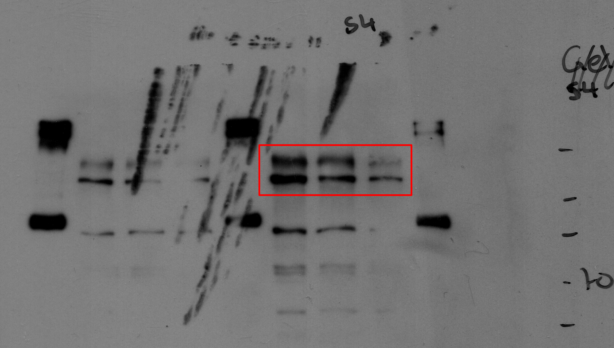

Supplement: Supplementary file 4 — Source Data [file 41467_2024_52875_MOESM4_ESM.zip › WB Full Scans/Figure_7/I/Fig.7_I_R115G-Plekhg5-HE.tif]

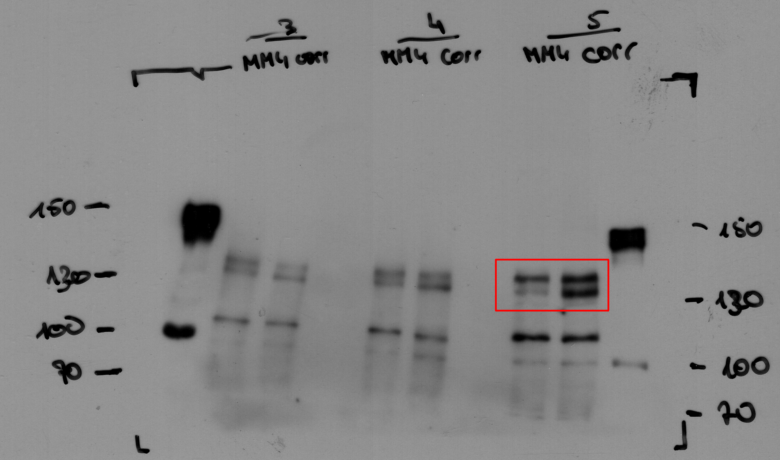

Supplement: Supplementary file 4 — Source Data [file 41467_2024_52875_MOESM4_ESM.zip › WB Full Scans/Figure_7/I/Fig.7_I_D90A-Plekhg5-HE.tif]

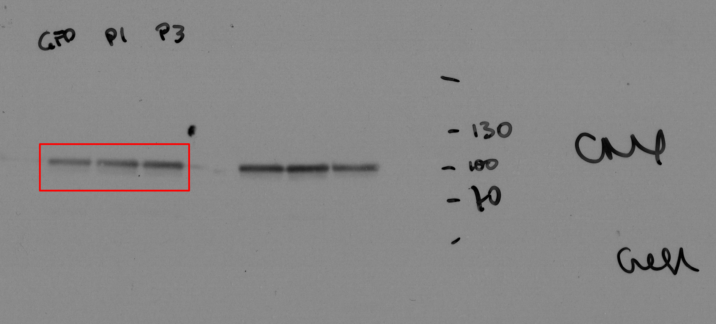

Supplement: Supplementary file 4 — Source Data [file 41467_2024_52875_MOESM4_ESM.zip › WB Full Scans/Figure_7/A/Fig.7_A_CANX.tif]

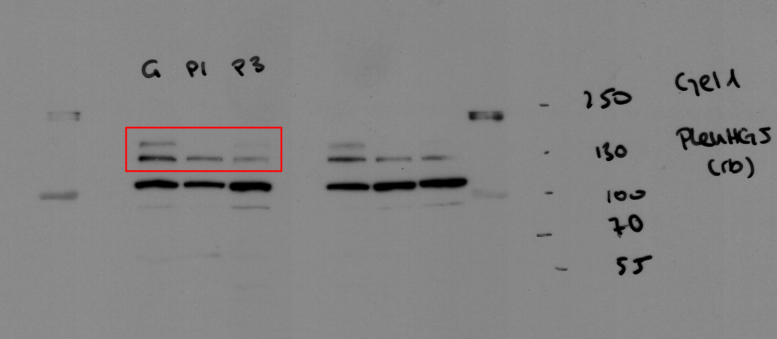

Supplement: Supplementary file 4 — Source Data [file 41467_2024_52875_MOESM4_ESM.zip › WB Full Scans/Figure_7/A/Fig.7_A_Plekhg5.tif]

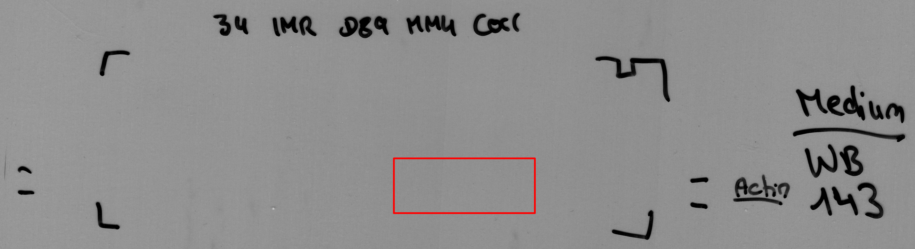

Supplement: Supplementary file 4 — Source Data [file 41467_2024_52875_MOESM4_ESM.zip › WB Full Scans/Figure_7/F/Fig.7_F_Actin-D90A_medium.tif]

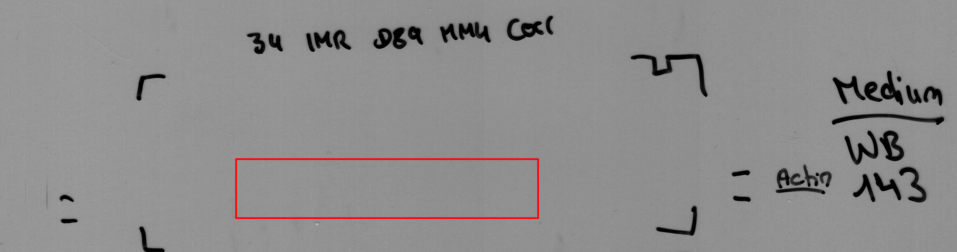

Supplement: Supplementary file 4 — Source Data [file 41467_2024_52875_MOESM4_ESM.zip › WB Full Scans/Figure_7/F/Fig.7_F_Actin_medium.tif]

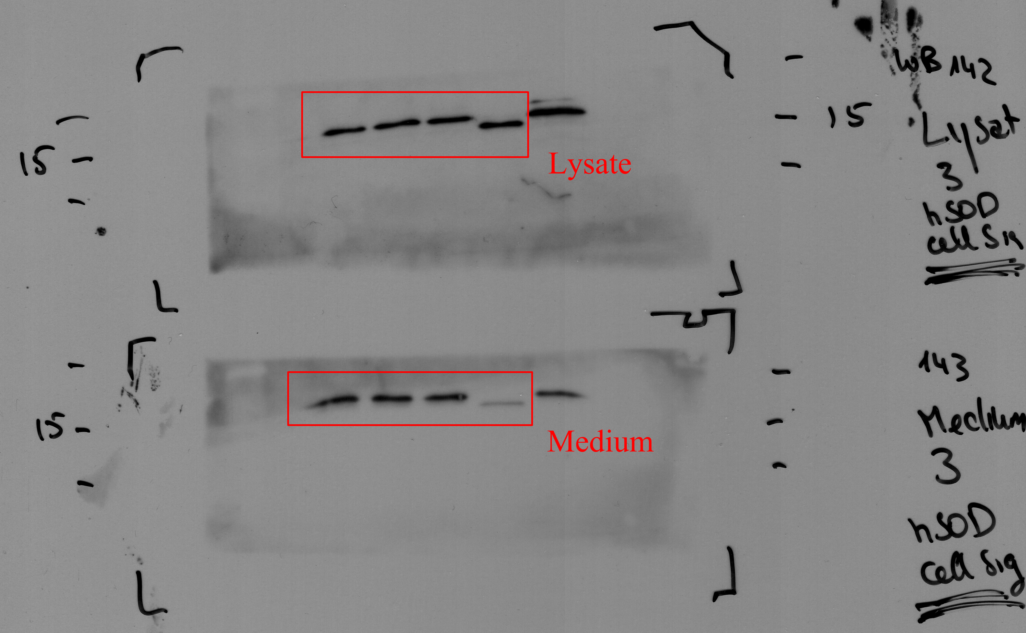

Supplement: Supplementary file 4 — Source Data [file 41467_2024_52875_MOESM4_ESM.zip › WB Full Scans/Figure_7/F/Fig.7_F_Sod1.tif]

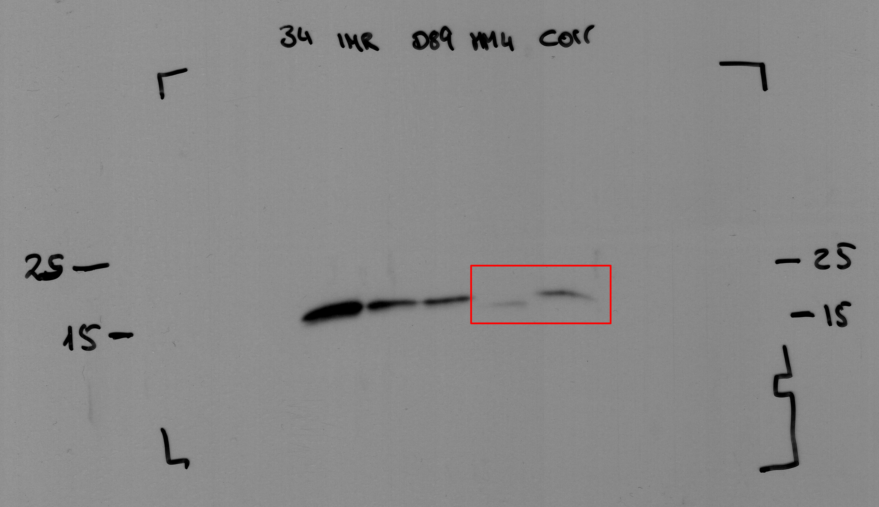

Supplement: Supplementary file 4 — Source Data [file 41467_2024_52875_MOESM4_ESM.zip › WB Full Scans/Figure_7/F/Fig.7_F_Sod1-D90A-lysate.tif]

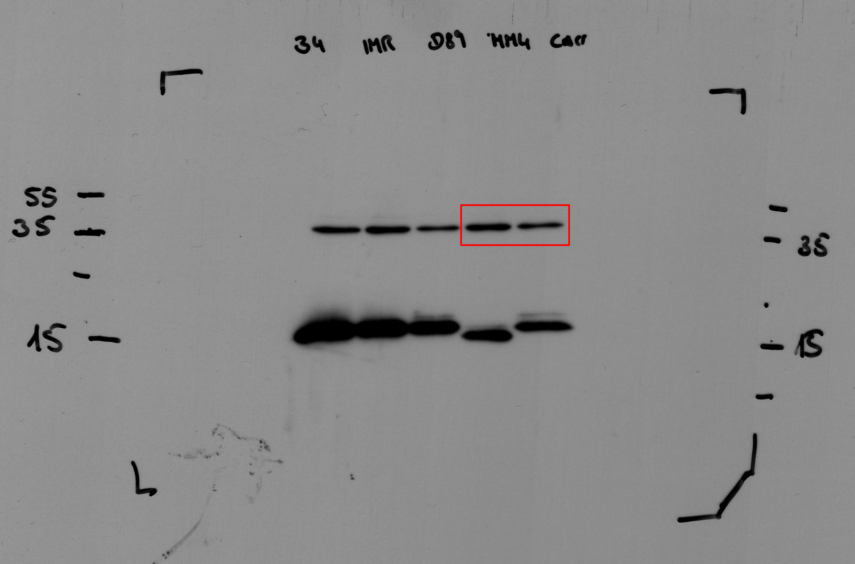

Supplement: Supplementary file 4 — Source Data [file 41467_2024_52875_MOESM4_ESM.zip › WB Full Scans/Figure_7/F/Fig.7_F_Actin-D90A-lysate.tif]

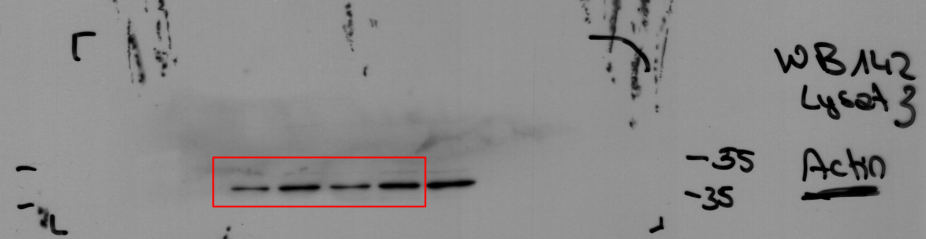

Supplement: Supplementary file 4 — Source Data [file 41467_2024_52875_MOESM4_ESM.zip › WB Full Scans/Figure_7/F/Fig.7_F_Actin_lysate.tif]

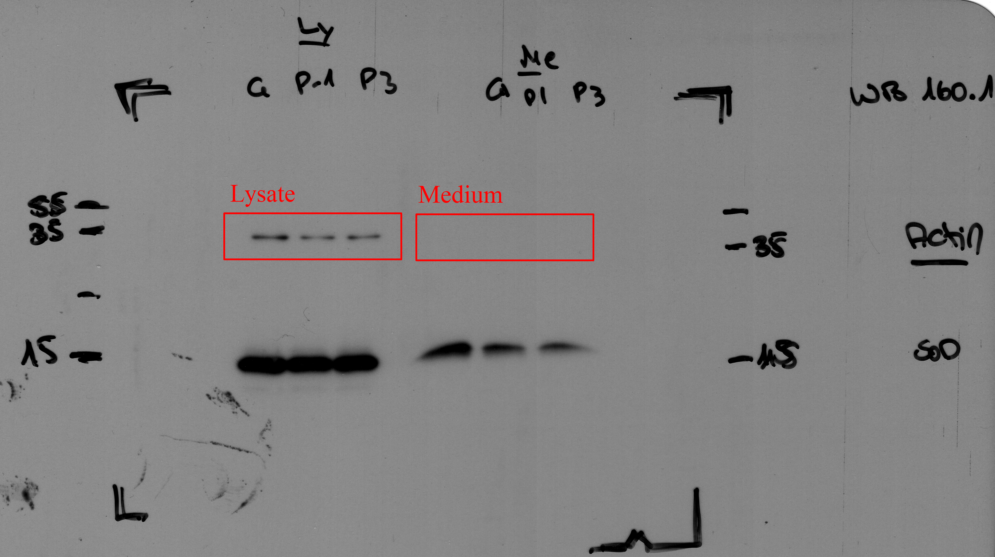

Supplement: Supplementary file 4 — Source Data [file 41467_2024_52875_MOESM4_ESM.zip › WB Full Scans/Figure_7/B/Fig.7_B_Actin.tif]

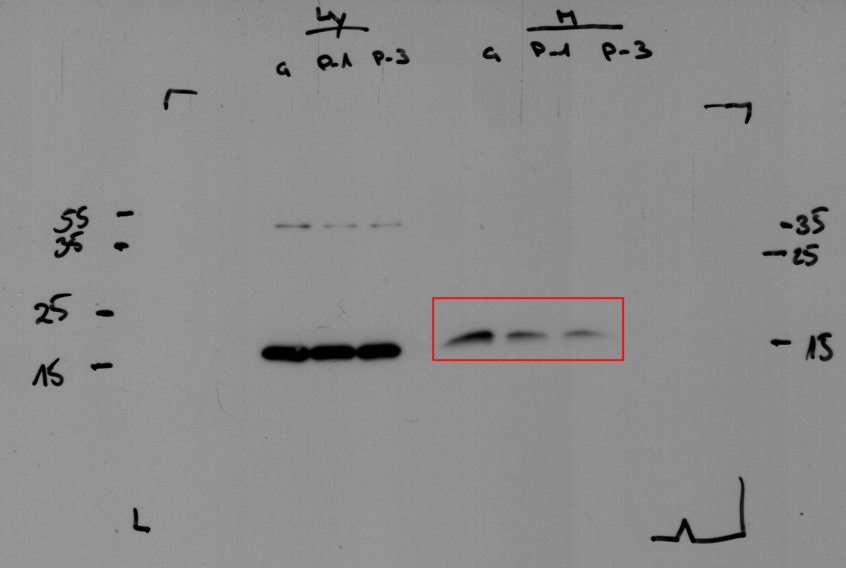

Supplement: Supplementary file 4 — Source Data [file 41467_2024_52875_MOESM4_ESM.zip › WB Full Scans/Figure_7/B/Fig.7_B_SOD1-medium.tif]

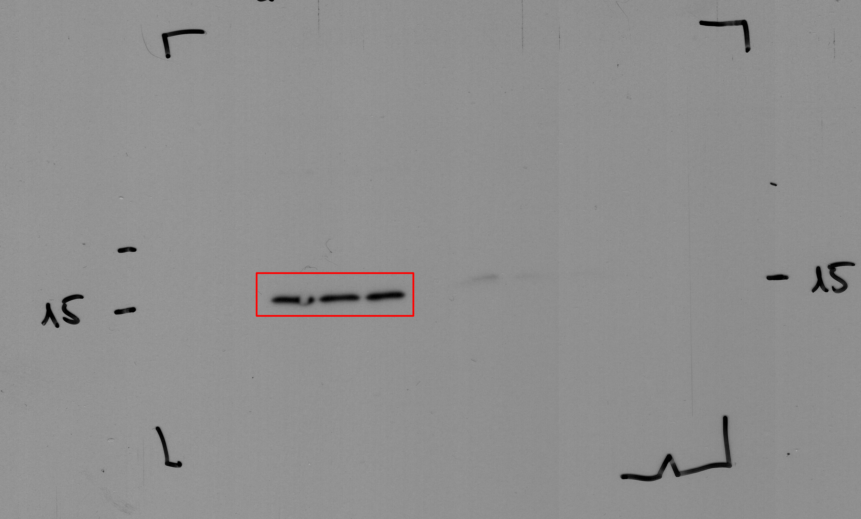

Supplement: Supplementary file 4 — Source Data [file 41467_2024_52875_MOESM4_ESM.zip › WB Full Scans/Figure_7/B/Fig.7_B_SOD1-lysate.tif]

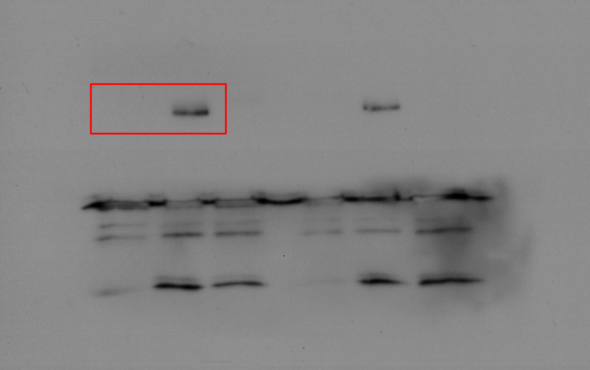

Supplement: Supplementary file 4 — Source Data [file 41467_2024_52875_MOESM4_ESM.zip › WB Full Scans/Figure_7/K/Fig.7_K_R115G-Lysate-Flag.tif]

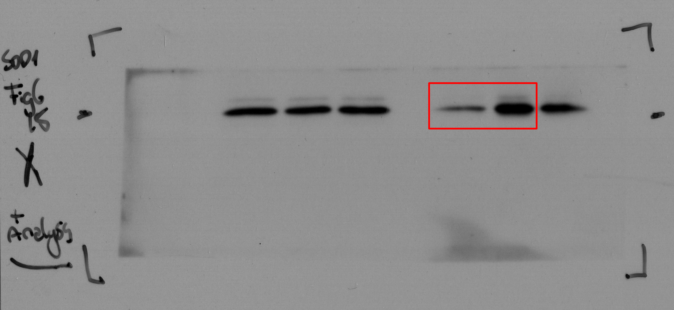

Supplement: Supplementary file 4 — Source Data [file 41467_2024_52875_MOESM4_ESM.zip › WB Full Scans/Figure_7/K/Fig.7_K_R115G-medium-Sod1.tif]

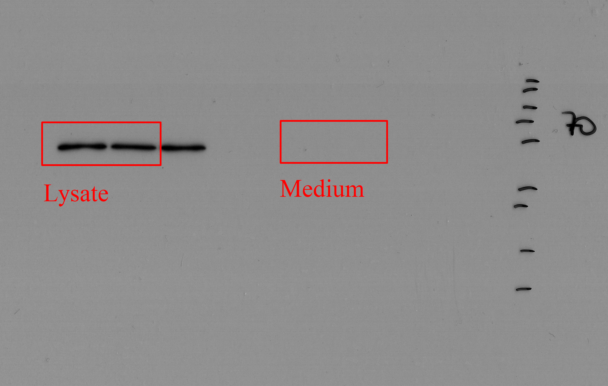

Supplement: Supplementary file 4 — Source Data [file 41467_2024_52875_MOESM4_ESM.zip › WB Full Scans/Figure_7/K/Fig.7_K_D90A-Tuj1.tif]

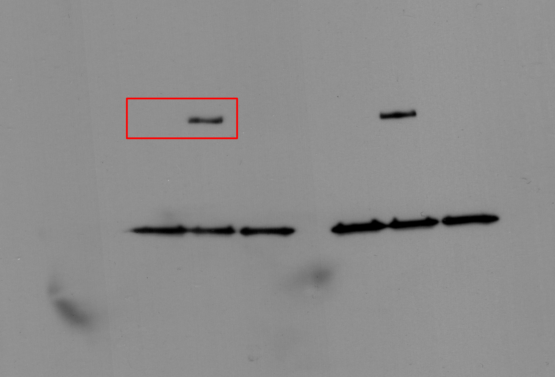

Supplement: Supplementary file 4 — Source Data [file 41467_2024_52875_MOESM4_ESM.zip › WB Full Scans/Figure_7/K/Fig.7_K_D90A-Lysate-Flag.tif]

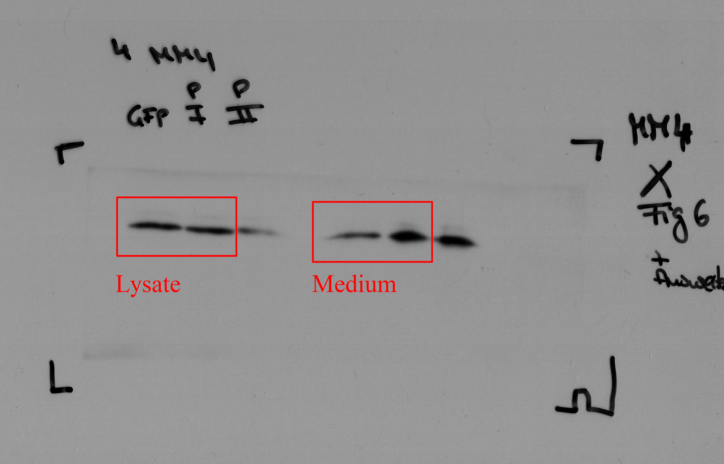

Supplement: Supplementary file 4 — Source Data [file 41467_2024_52875_MOESM4_ESM.zip › WB Full Scans/Figure_7/K/Fig.7_K_D90A-Sod1.tif]

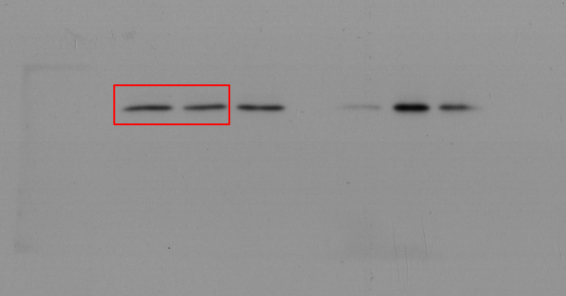

Supplement: Supplementary file 4 — Source Data [file 41467_2024_52875_MOESM4_ESM.zip › WB Full Scans/Figure_7/K/Fig.7_K_R115G-lysate-Sod1.tif]

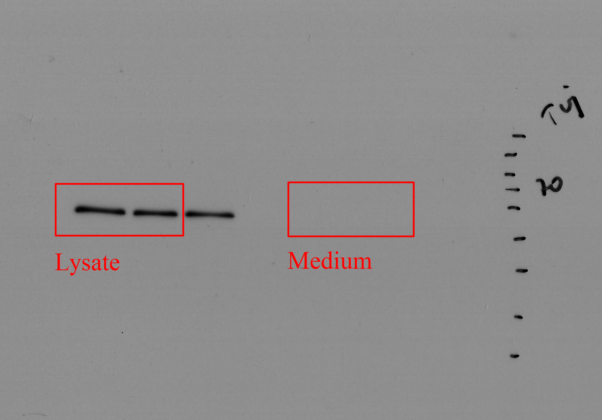

Supplement: Supplementary file 4 — Source Data [file 41467_2024_52875_MOESM4_ESM.zip › WB Full Scans/Figure_7/K/Fig.7_K_R115G-Tuj1.tif]

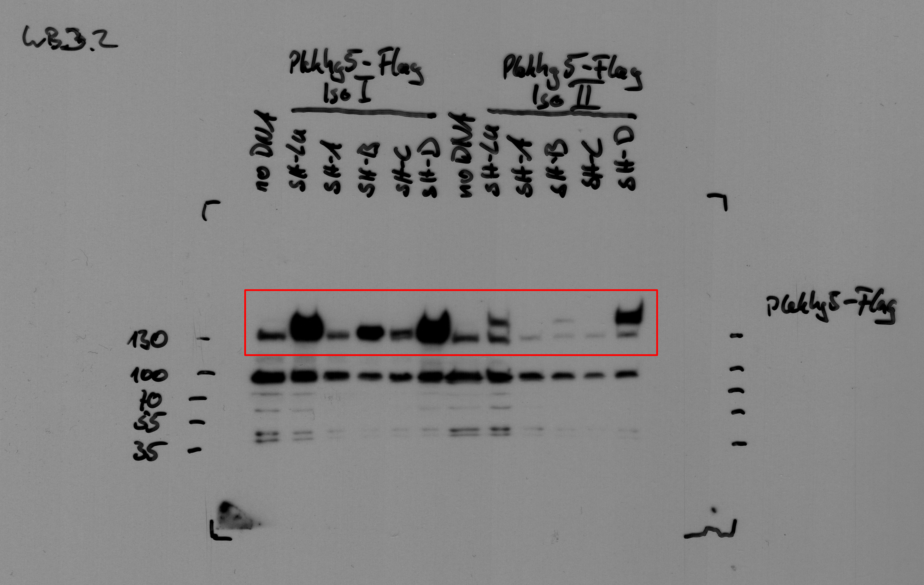

Supplement: Supplementary file 4 — Source Data [file 41467_2024_52875_MOESM4_ESM.zip › WB Full Scans/Supplementary_Figure_4/A/Sup.Fig.4_A_Flag-HE.tif]

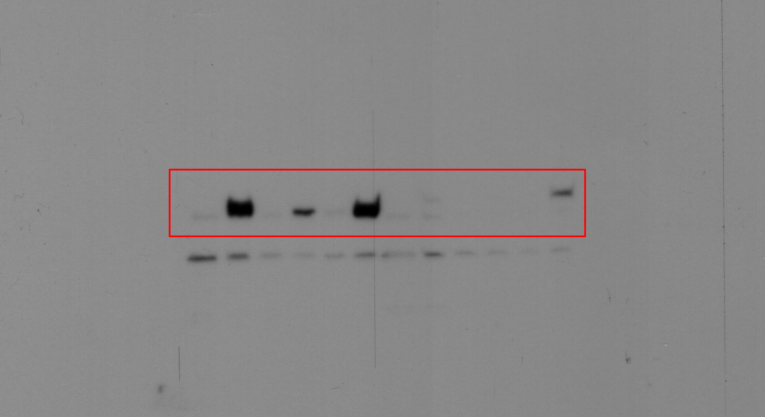

Supplement: Supplementary file 4 — Source Data [file 41467_2024_52875_MOESM4_ESM.zip › WB Full Scans/Supplementary_Figure_4/A/Sup.Fig.4_A_Flag-LE.tif]

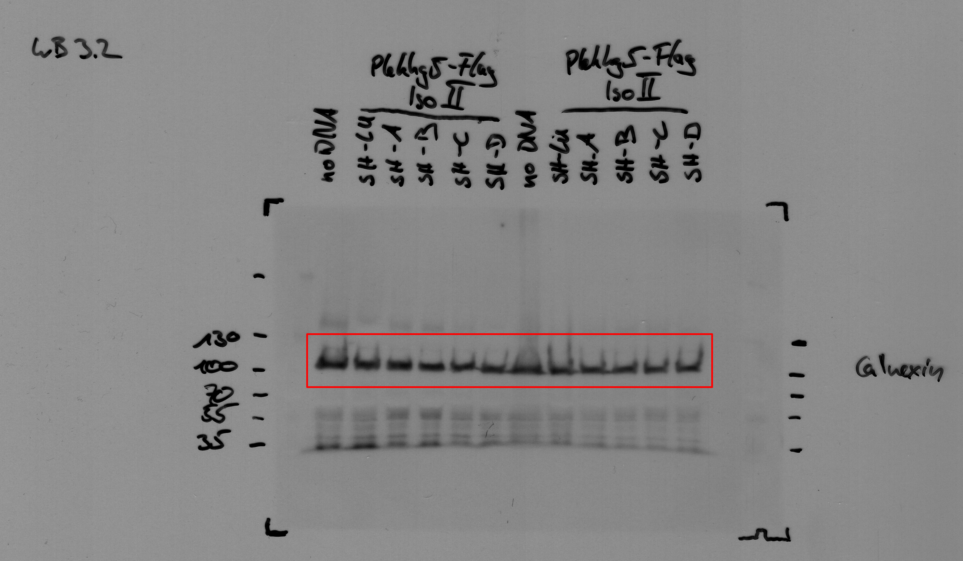

Supplement: Supplementary file 4 — Source Data [file 41467_2024_52875_MOESM4_ESM.zip › WB Full Scans/Supplementary_Figure_4/A/Sup.Fig.4_A_Calnexin.tif]

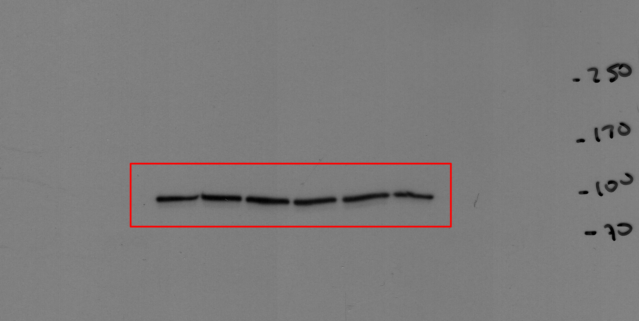

Supplement: Supplementary file 4 — Source Data [file 41467_2024_52875_MOESM4_ESM.zip › WB Full Scans/Supplementary_Figure_4/D/Sup.Fig.4_D_Calnexin.tif]

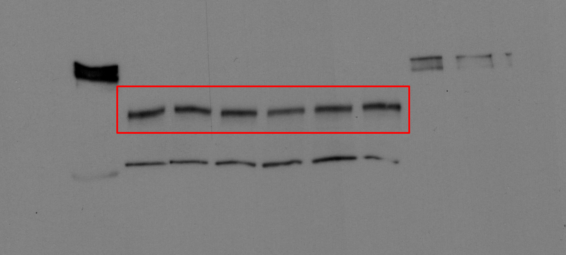

Supplement: Supplementary file 4 — Source Data [file 41467_2024_52875_MOESM4_ESM.zip › WB Full Scans/Supplementary_Figure_4/D/Sup.Fig.4_D_Plekhg5.tif]

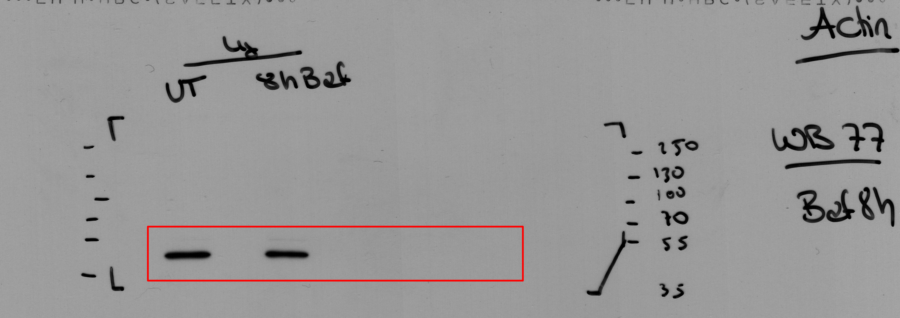

Supplement: Supplementary file 4 — Source Data [file 41467_2024_52875_MOESM4_ESM.zip › WB Full Scans/Figure_5/A/Fig.5_A_Actin.tif]

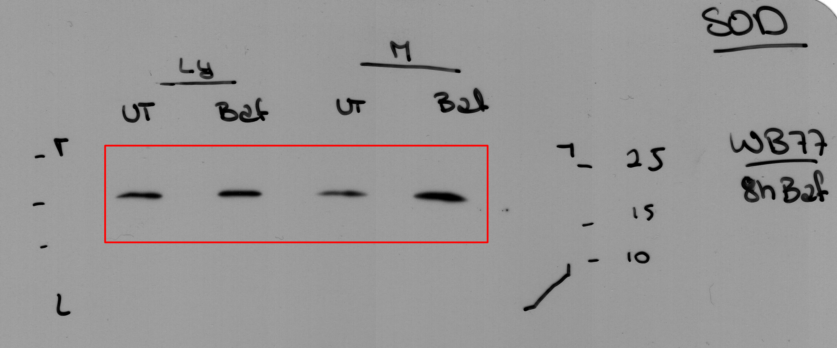

Supplement: Supplementary file 4 — Source Data [file 41467_2024_52875_MOESM4_ESM.zip › WB Full Scans/Figure_5/A/Fig.5_A_Sod1.tif]

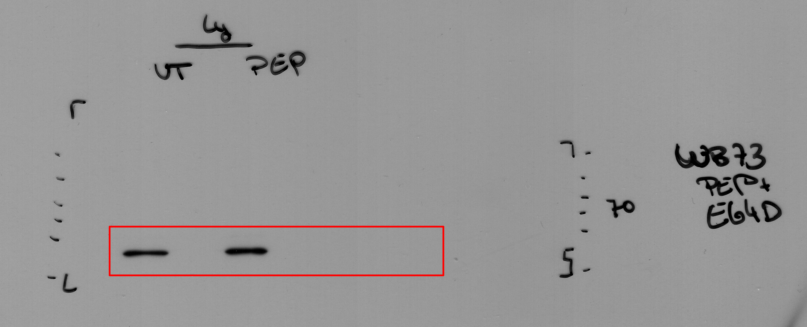

Supplement: Supplementary file 4 — Source Data [file 41467_2024_52875_MOESM4_ESM.zip › WB Full Scans/Figure_5/B/Fig.5_B_Actin.tif]

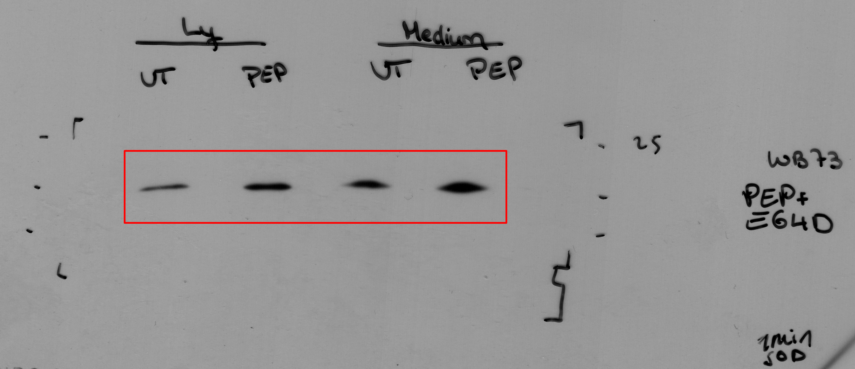

Supplement: Supplementary file 4 — Source Data [file 41467_2024_52875_MOESM4_ESM.zip › WB Full Scans/Figure_5/B/Fig.5_B_Sod1.tif]

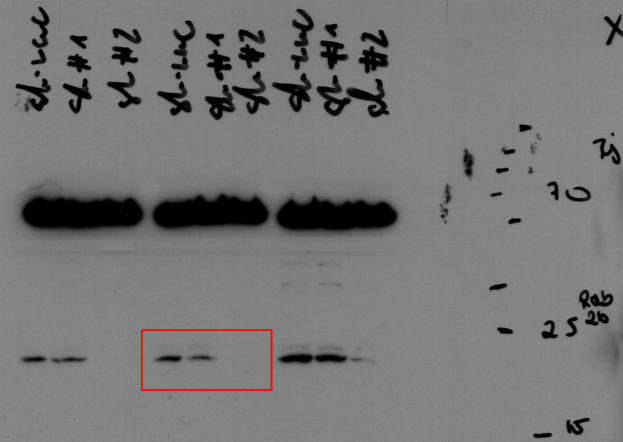

Supplement: Supplementary file 4 — Source Data [file 41467_2024_52875_MOESM4_ESM.zip › WB Full Scans/Figure_2/I/Fig.2_I_Rab26.tif]

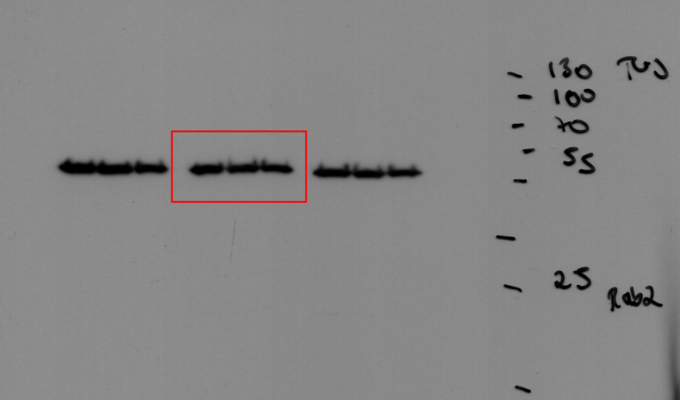

Supplement: Supplementary file 4 — Source Data [file 41467_2024_52875_MOESM4_ESM.zip › WB Full Scans/Figure_2/I/Fig.2_I_Tuj1.tif]

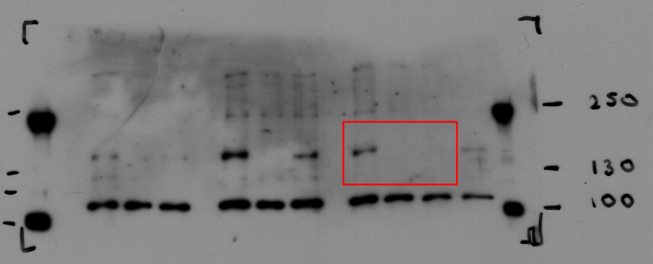

Supplement: Supplementary file 4 — Source Data [file 41467_2024_52875_MOESM4_ESM.zip › WB Full Scans/Figure_2/A/Fig.2_A_Plekhg5.tif]

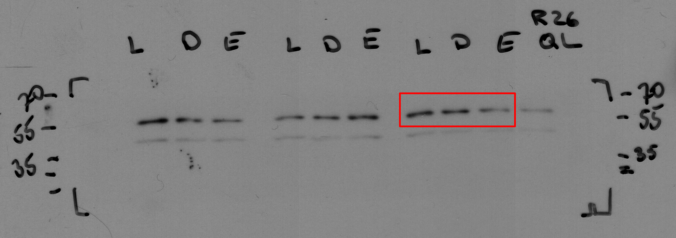

Supplement: Supplementary file 4 — Source Data [file 41467_2024_52875_MOESM4_ESM.zip › WB Full Scans/Figure_2/A/Fig.2_A_Tuj1.tif]

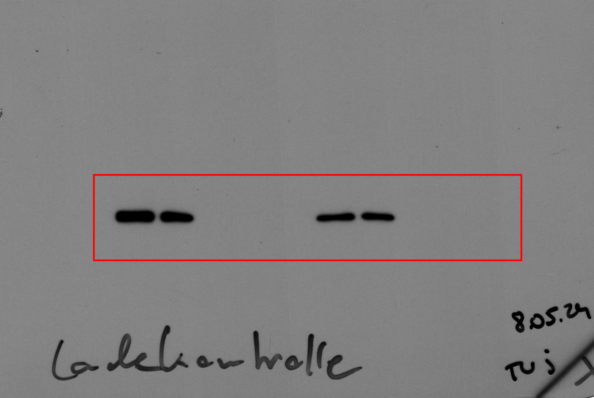

Supplement: Supplementary file 4 — Source Data [file 41467_2024_52875_MOESM4_ESM.zip › WB Full Scans/Figure_2/F/Fig.2_F_Tuj1.tif]

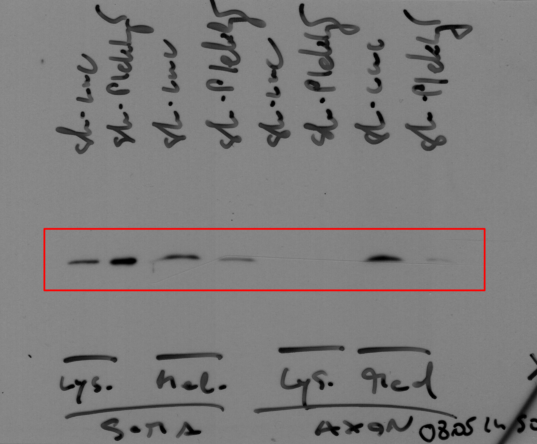

Supplement: Supplementary file 4 — Source Data [file 41467_2024_52875_MOESM4_ESM.zip › WB Full Scans/Figure_2/F/Fig.2_F_Sod1_LE.tif]

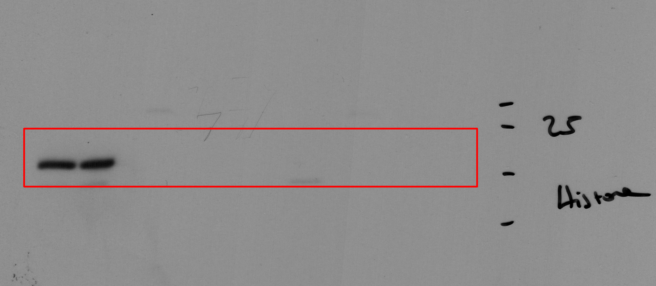

Supplement: Supplementary file 4 — Source Data [file 41467_2024_52875_MOESM4_ESM.zip › WB Full Scans/Figure_2/F/Fig.2_F_Histon.tif]

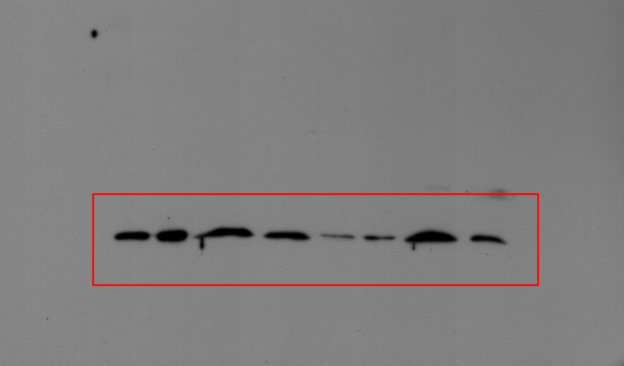

Supplement: Supplementary file 4 — Source Data [file 41467_2024_52875_MOESM4_ESM.zip › WB Full Scans/Figure_2/F/Fig.2_F_Sod1_HE.tif]

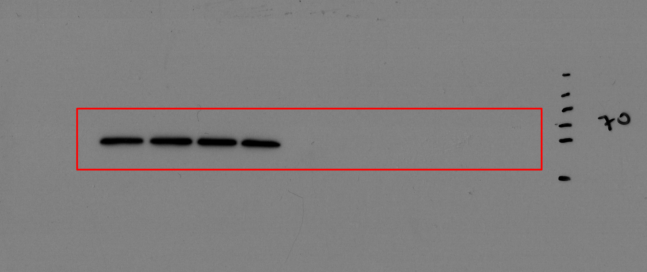

Supplement: Supplementary file 4 — Source Data [file 41467_2024_52875_MOESM4_ESM.zip › WB Full Scans/Figure_2/M/Fig.2_M_Tuj1.tif]

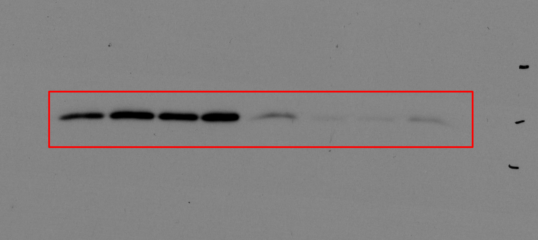

Supplement: Supplementary file 4 — Source Data [file 41467_2024_52875_MOESM4_ESM.zip › WB Full Scans/Figure_2/M/Fig.2_M_Sod1.tif]

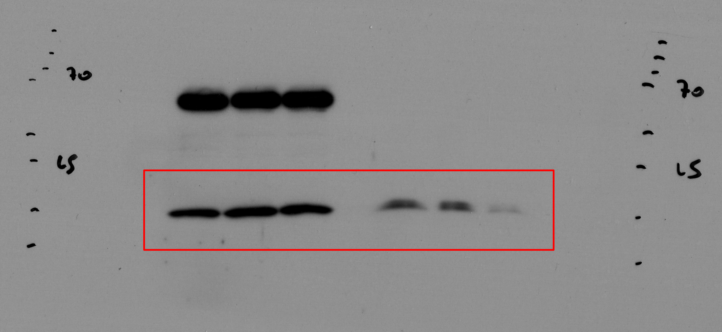

Supplement: Supplementary file 4 — Source Data [file 41467_2024_52875_MOESM4_ESM.zip › WB Full Scans/Figure_2/J/Fig.2_J_Sod1.tif]

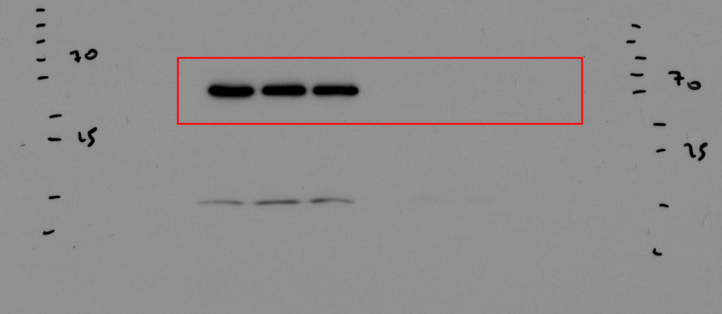

Supplement: Supplementary file 4 — Source Data [file 41467_2024_52875_MOESM4_ESM.zip › WB Full Scans/Figure_2/J/Fig.2_J_Tuj1.tif]

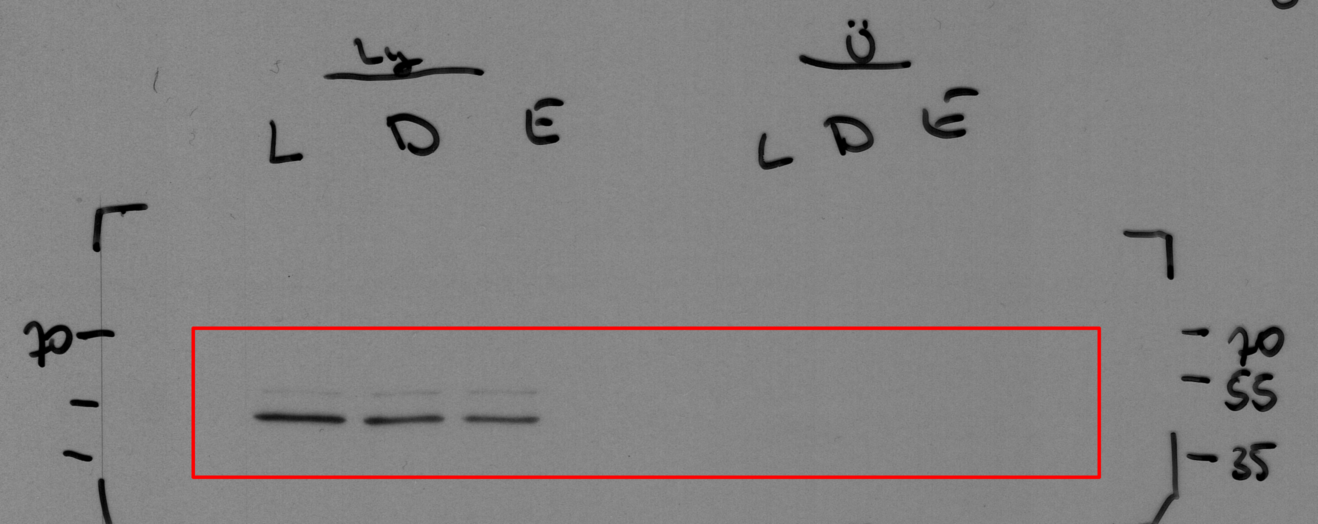

Supplement: Supplementary file 4 — Source Data [file 41467_2024_52875_MOESM4_ESM.zip › WB Full Scans/Figure_2/D/Fig.2_D_Actin.tif]

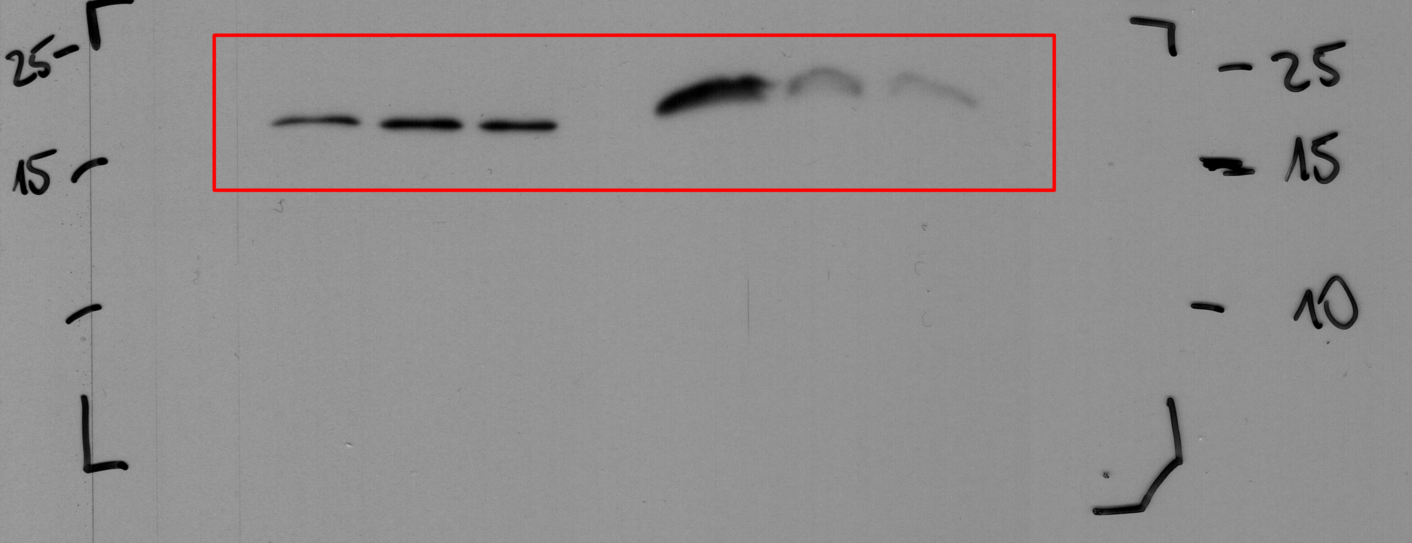

Supplement: Supplementary file 4 — Source Data [file 41467_2024_52875_MOESM4_ESM.zip › WB Full Scans/Figure_2/D/Fig.2_D_Sod1.tif]

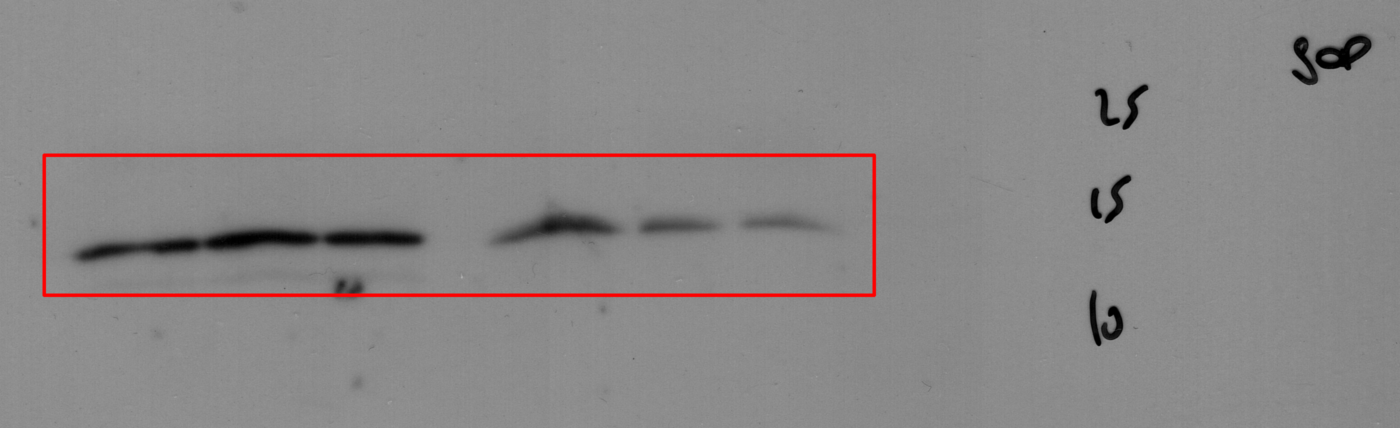

Supplement: Supplementary file 4 — Source Data [file 41467_2024_52875_MOESM4_ESM.zip › WB Full Scans/Figure_2/B/Fig.2_B_Sod1.tif]

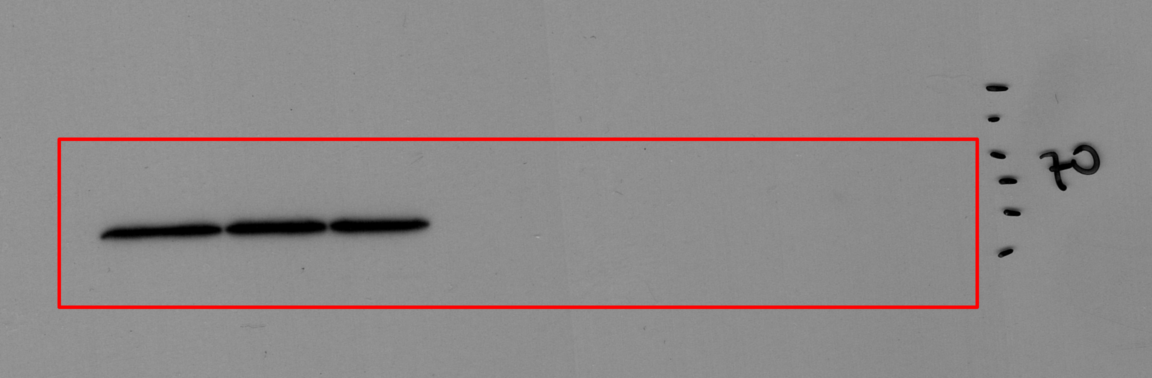

Supplement: Supplementary file 4 — Source Data [file 41467_2024_52875_MOESM4_ESM.zip › WB Full Scans/Figure_2/B/Fig.2_B_Tuj1.tif]

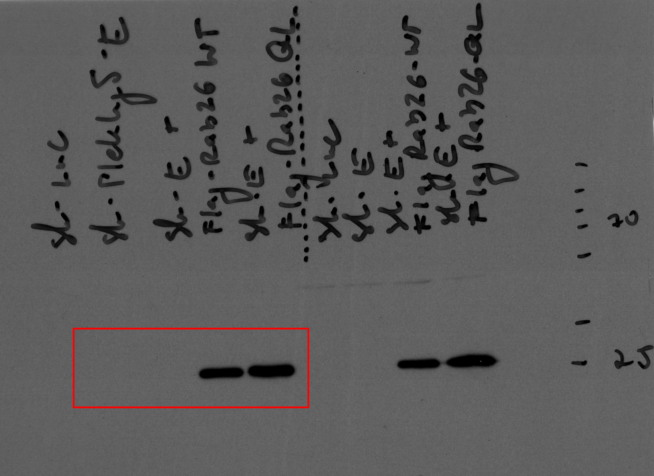

Supplement: Supplementary file 4 — Source Data [file 41467_2024_52875_MOESM4_ESM.zip › WB Full Scans/Figure_2/L/Fig.2_L_Flag.tif]

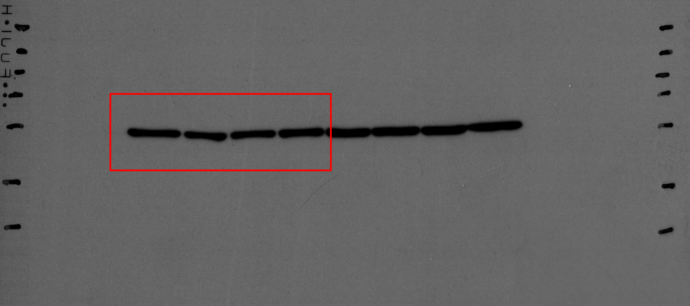

Supplement: Supplementary file 4 — Source Data [file 41467_2024_52875_MOESM4_ESM.zip › WB Full Scans/Figure_2/L/Fig.2_L_Tuj1.tif]

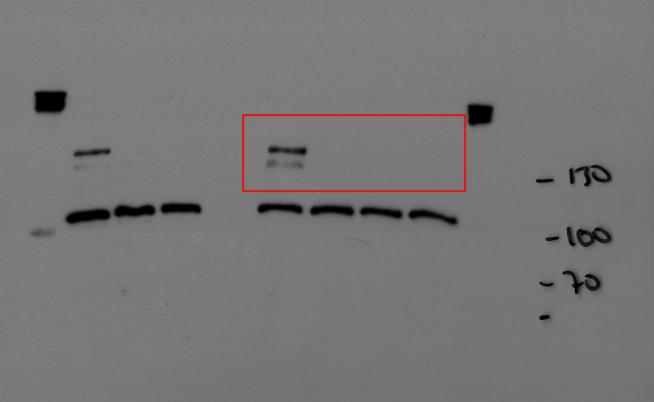

Supplement: Supplementary file 4 — Source Data [file 41467_2024_52875_MOESM4_ESM.zip › WB Full Scans/Figure_2/L/Fig.2_L_Plekhg5.tif]

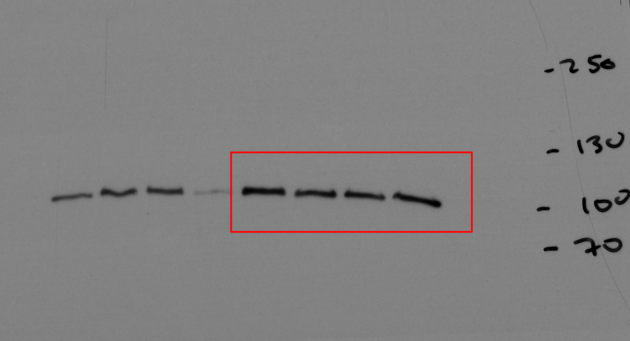

Supplement: Supplementary file 4 — Source Data [file 41467_2024_52875_MOESM4_ESM.zip › WB Full Scans/Figure_2/L/Fig.2_L_Calnexin.tif]

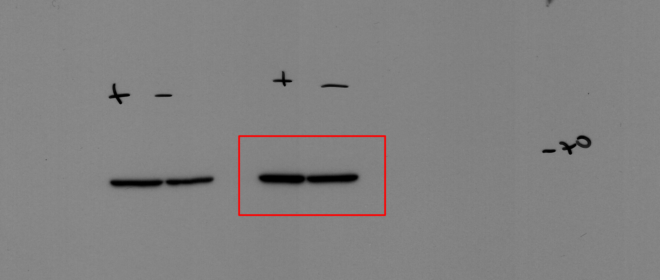

Supplement: Supplementary file 4 — Source Data [file 41467_2024_52875_MOESM4_ESM.zip › WB Full Scans/Figure_3/I/Fig.3_I_Tuj1.tif]

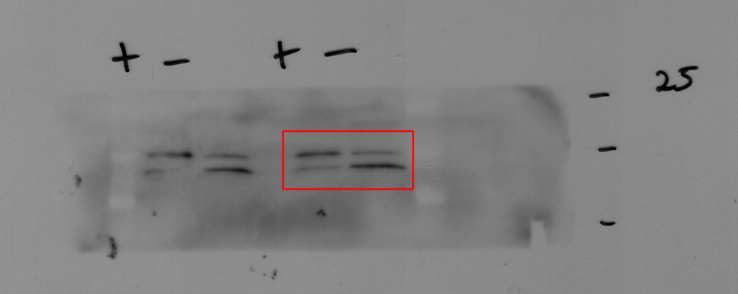

Supplement: Supplementary file 4 — Source Data [file 41467_2024_52875_MOESM4_ESM.zip › WB Full Scans/Figure_3/I/Fig.3_I_LC3.tif]

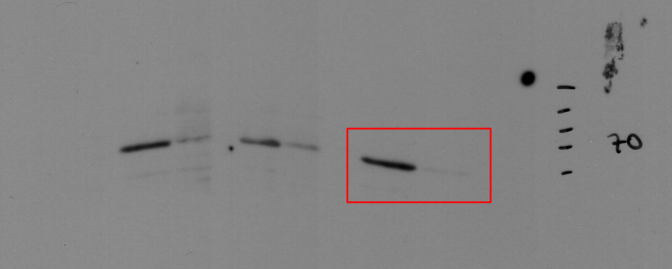

Supplement: Supplementary file 4 — Source Data [file 41467_2024_52875_MOESM4_ESM.zip › WB Full Scans/Figure_3/I/Fig.3_I_Atg5-Atg12.tif]

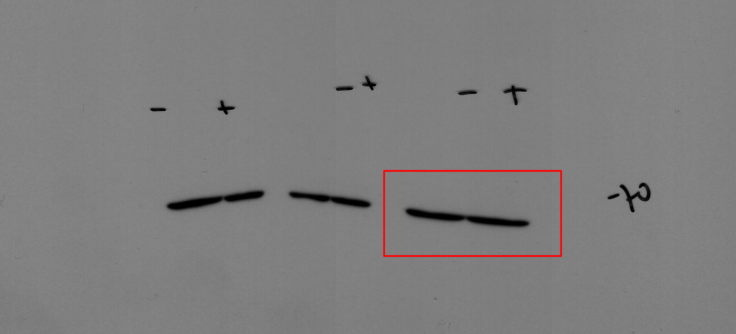

Supplement: Supplementary file 4 — Source Data [file 41467_2024_52875_MOESM4_ESM.zip › WB Full Scans/Figure_3/I/Fig.3_I_Actin.tif]

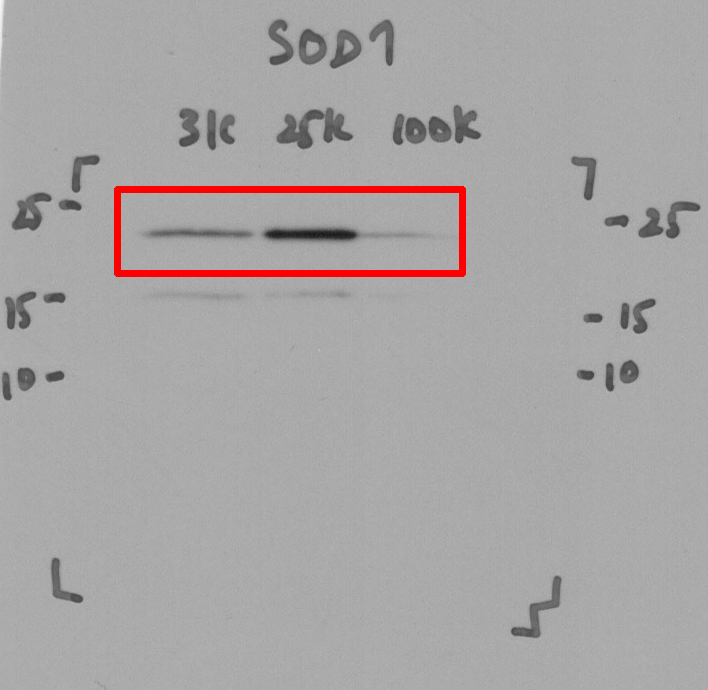

Supplement: Supplementary file 4 — Source Data [file 41467_2024_52875_MOESM4_ESM.zip › WB Full Scans/Figure_3/N/Fig.3_N_Sod1.tif]

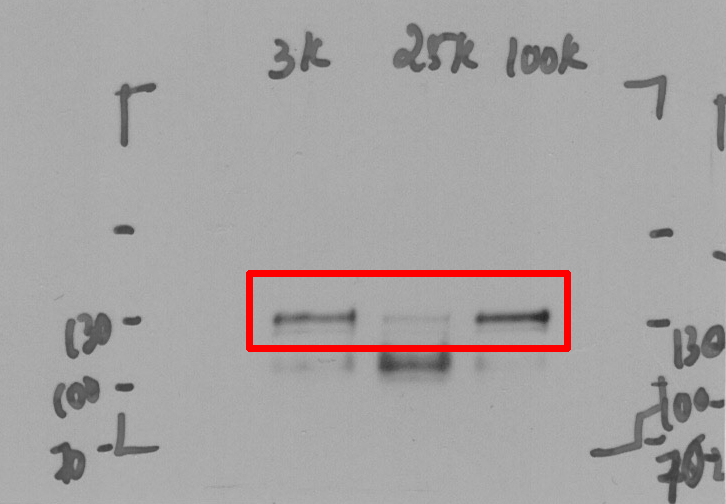

Supplement: Supplementary file 4 — Source Data [file 41467_2024_52875_MOESM4_ESM.zip › WB Full Scans/Figure_3/N/Fig.3_N_GM130.tif]

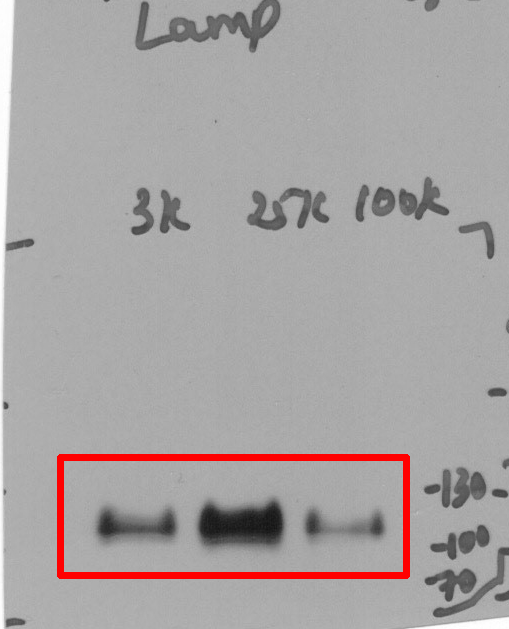

Supplement: Supplementary file 4 — Source Data [file 41467_2024_52875_MOESM4_ESM.zip › WB Full Scans/Figure_3/N/Fig.3_N_Lamp1.tif]

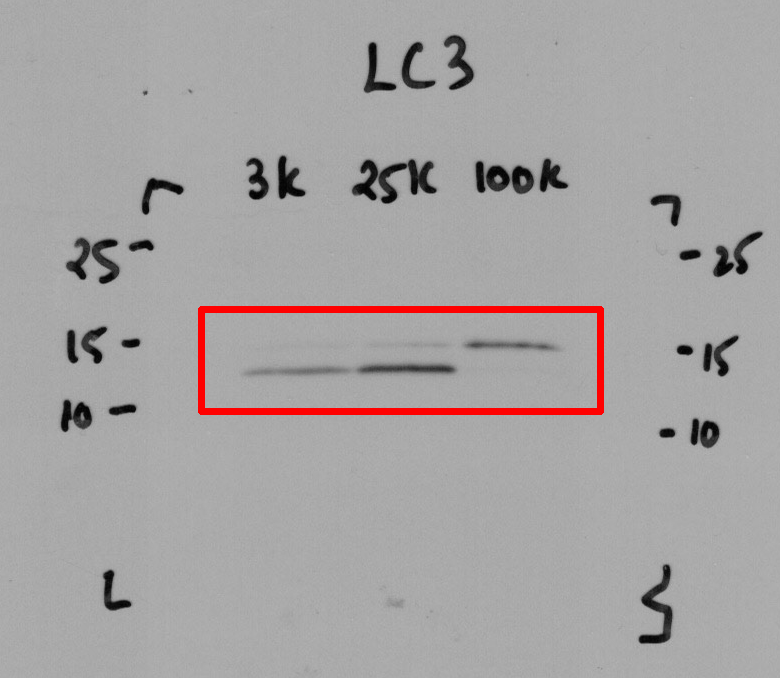

Supplement: Supplementary file 4 — Source Data [file 41467_2024_52875_MOESM4_ESM.zip › WB Full Scans/Figure_3/N/Fig.3_N_LC3.tif]

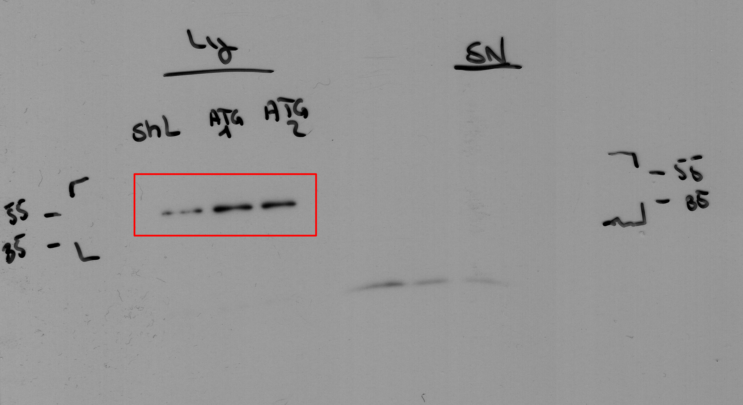

Supplement: Supplementary file 4 — Source Data [file 41467_2024_52875_MOESM4_ESM.zip › WB Full Scans/Figure_3/G/Fig.3_G_Actin.tif]

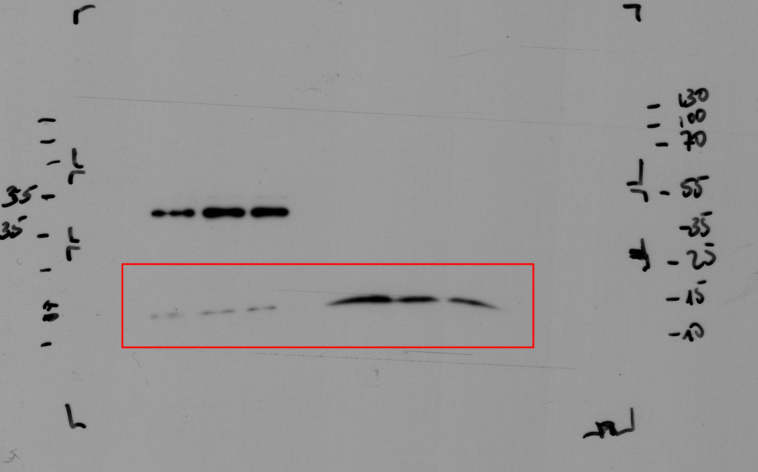

Supplement: Supplementary file 4 — Source Data [file 41467_2024_52875_MOESM4_ESM.zip › WB Full Scans/Figure_3/G/Fig.3_G_Sod1.tif]

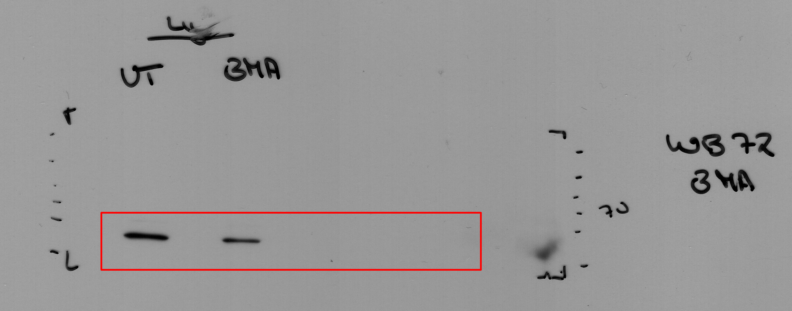

Supplement: Supplementary file 4 — Source Data [file 41467_2024_52875_MOESM4_ESM.zip › WB Full Scans/Figure_3/A/Fig.3_A_Actin.tif]

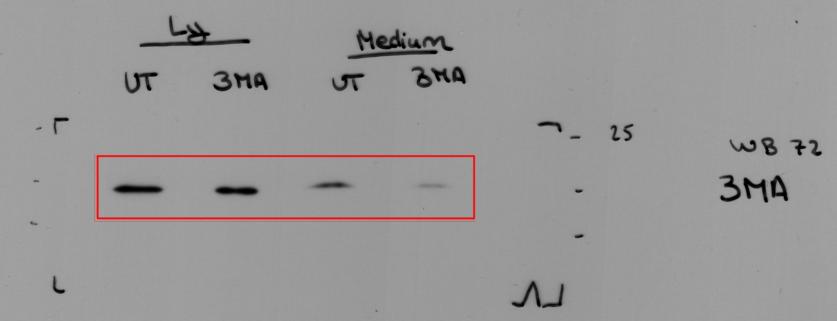

Supplement: Supplementary file 4 — Source Data [file 41467_2024_52875_MOESM4_ESM.zip › WB Full Scans/Figure_3/A/Fig.3_A_Sod1.tif]

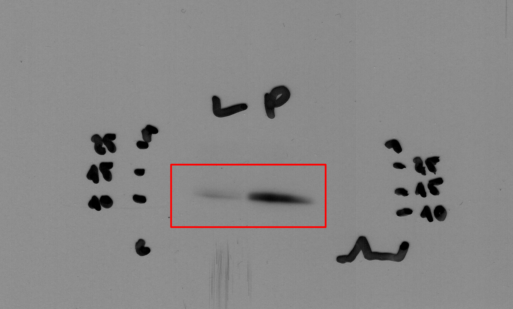

Supplement: Supplementary file 4 — Source Data [file 41467_2024_52875_MOESM4_ESM.zip › WB Full Scans/Figure_3/O/Fig.3_O_CytoC.tif]

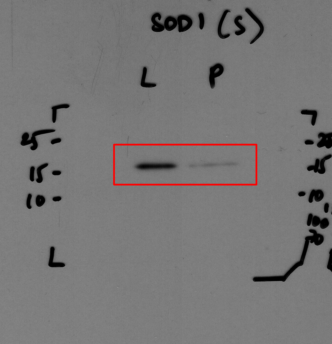

Supplement: Supplementary file 4 — Source Data [file 41467_2024_52875_MOESM4_ESM.zip › WB Full Scans/Figure_3/O/Fig.3_O_Sod1.tif]

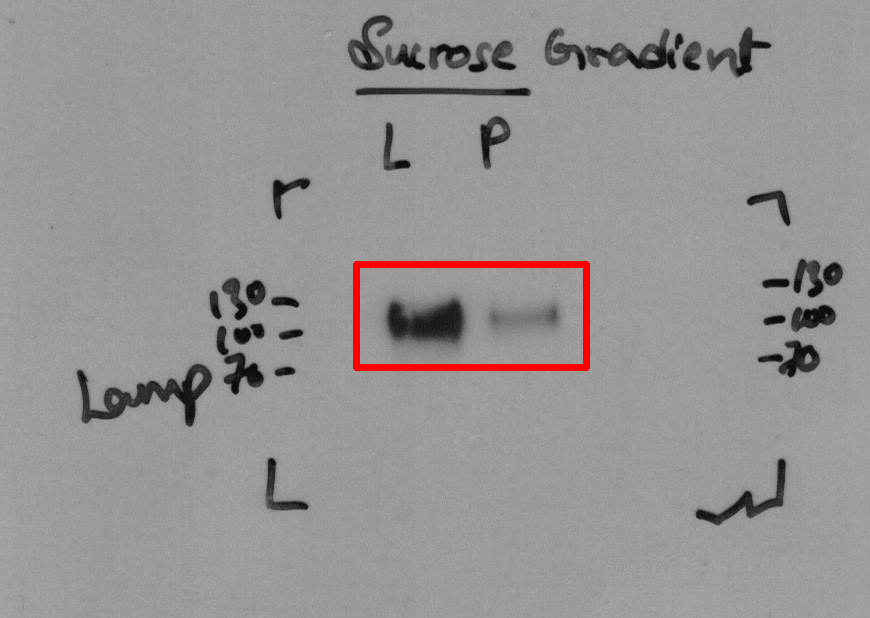

Supplement: Supplementary file 4 — Source Data [file 41467_2024_52875_MOESM4_ESM.zip › WB Full Scans/Figure_3/O/Fig.3_O_Lamp1.tif]

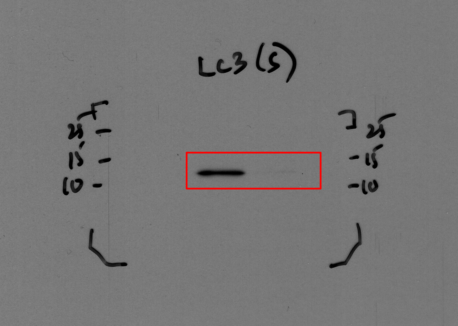

Supplement: Supplementary file 4 — Source Data [file 41467_2024_52875_MOESM4_ESM.zip › WB Full Scans/Figure_3/O/Fig.3_O_LC3.tif]

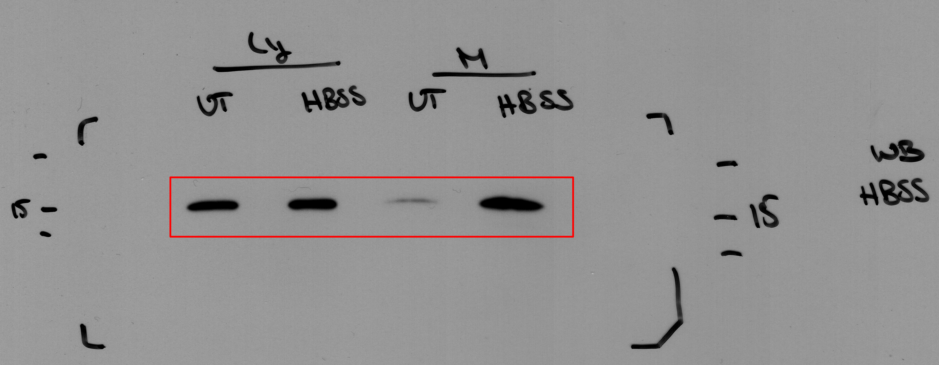

Supplement: Supplementary file 4 — Source Data [file 41467_2024_52875_MOESM4_ESM.zip › WB Full Scans/Figure_3/D/Fig.3_D_Sod1.tif]

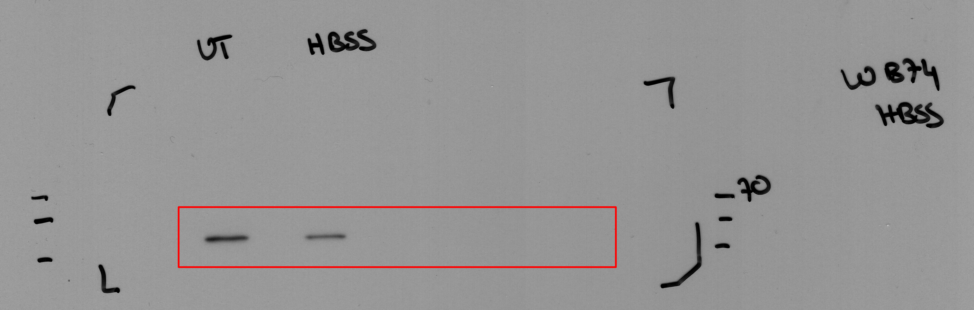

Supplement: Supplementary file 4 — Source Data [file 41467_2024_52875_MOESM4_ESM.zip › WB Full Scans/Figure_3/D/Fig.3_D_Actin.tif]

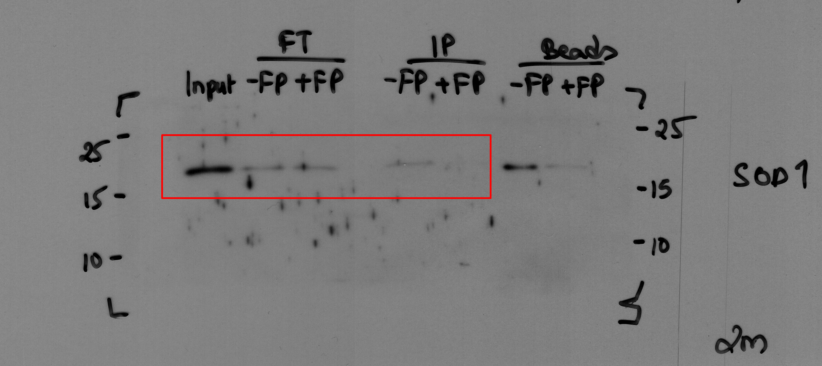

Supplement: Supplementary file 4 — Source Data [file 41467_2024_52875_MOESM4_ESM.zip › WB Full Scans/Figure_3/Q/Fig.3_Q_Sod1.tif]

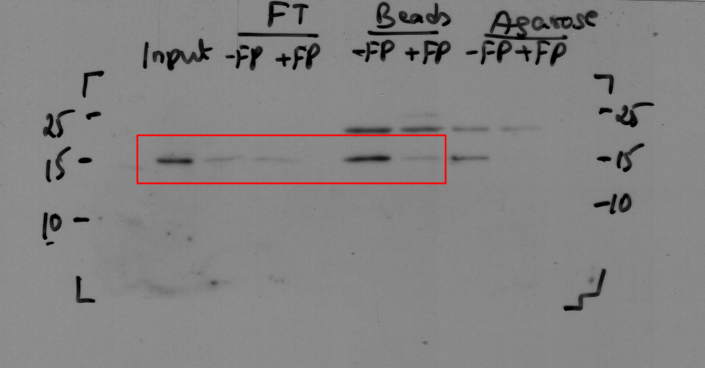

Supplement: Supplementary file 4 — Source Data [file 41467_2024_52875_MOESM4_ESM.zip › WB Full Scans/Figure_3/Q/Fig.3_Q_Flag-LC3.tif]

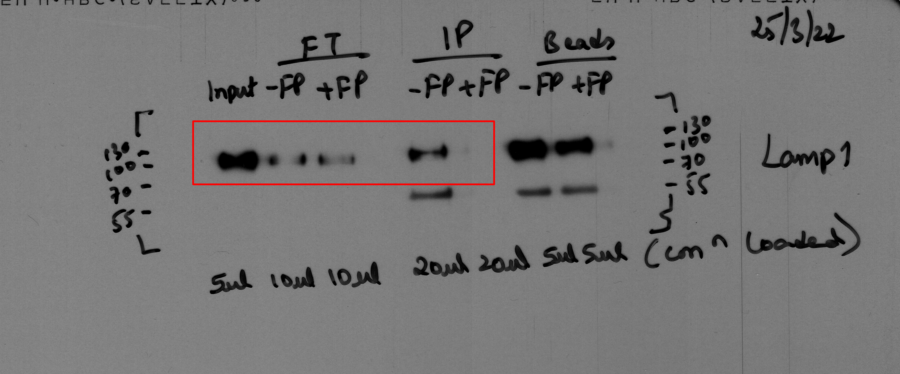

Supplement: Supplementary file 4 — Source Data [file 41467_2024_52875_MOESM4_ESM.zip › WB Full Scans/Figure_3/Q/Fig.3_Q_Lamp1.tif]

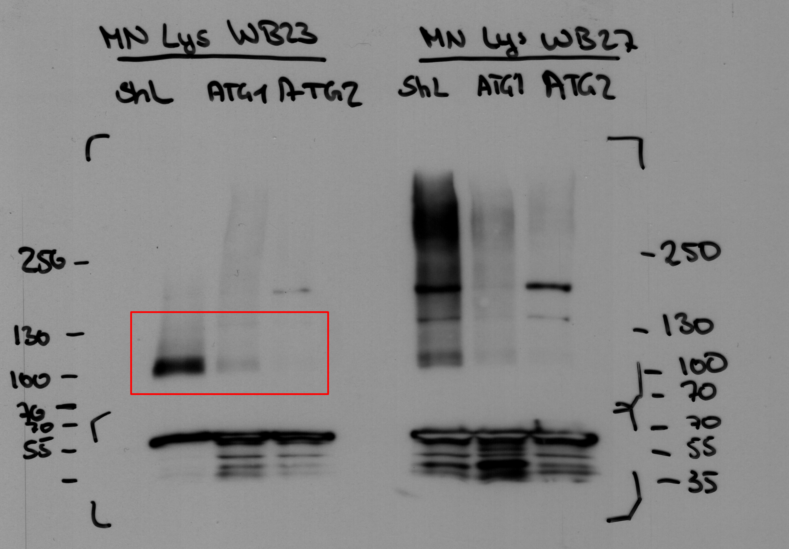

Supplement: Supplementary file 4 — Source Data [file 41467_2024_52875_MOESM4_ESM.zip › WB Full Scans/Figure_3/E/Fig.3_E_Atg9.tif]

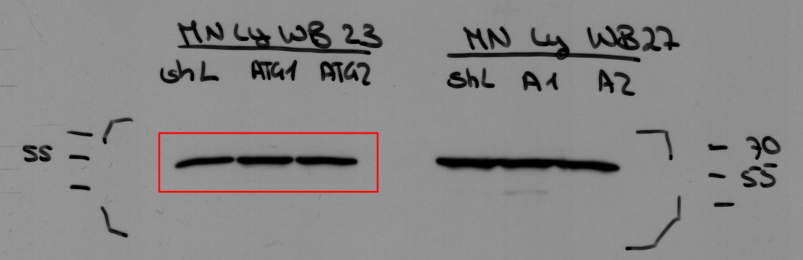

Supplement: Supplementary file 4 — Source Data [file 41467_2024_52875_MOESM4_ESM.zip › WB Full Scans/Figure_3/E/Fig.3_E_Tuj1.tif]

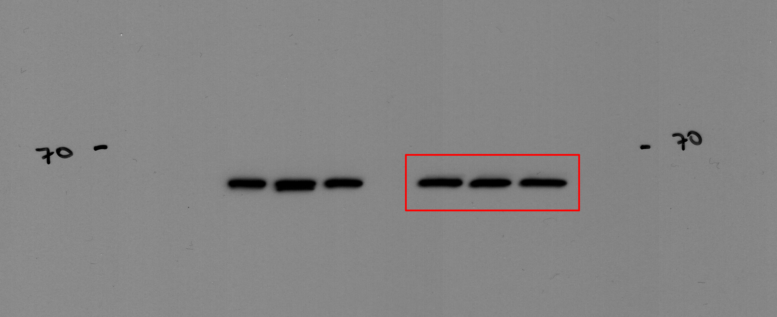

Supplement: Supplementary file 4 — Source Data [file 41467_2024_52875_MOESM4_ESM.zip › WB Full Scans/Figure_3/E/Fig.3_E_Actin.tif]

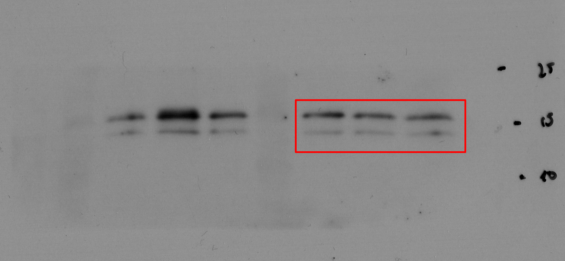

Supplement: Supplementary file 4 — Source Data [file 41467_2024_52875_MOESM4_ESM.zip › WB Full Scans/Figure_3/E/Fig.3_E_LC3.tif]

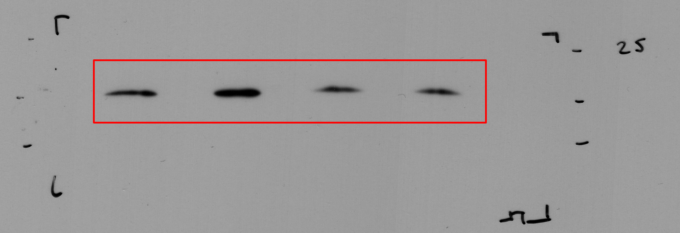

Supplement: Supplementary file 4 — Source Data [file 41467_2024_52875_MOESM4_ESM.zip › WB Full Scans/Figure_3/B/Fig.3_B_Sod1.tif]

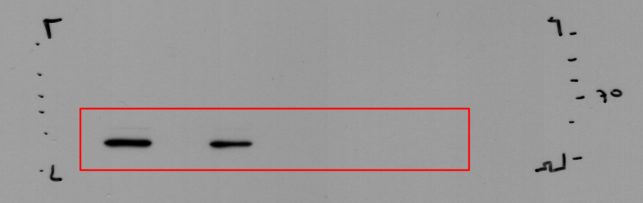

Supplement: Supplementary file 4 — Source Data [file 41467_2024_52875_MOESM4_ESM.zip › WB Full Scans/Figure_3/B/Fig.3_B_Actin.tif]

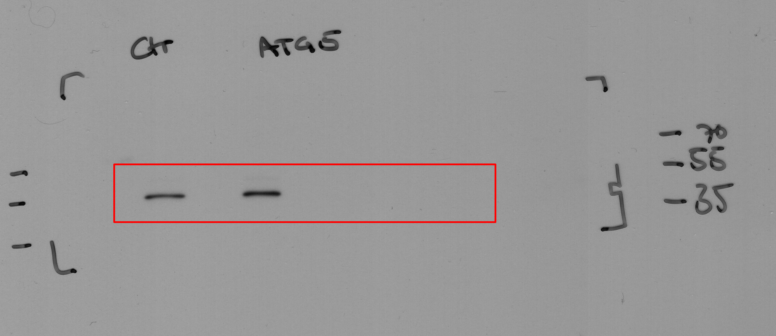

Supplement: Supplementary file 4 — Source Data [file 41467_2024_52875_MOESM4_ESM.zip › WB Full Scans/Figure_3/K/Fig.3_K_Actin.tif]

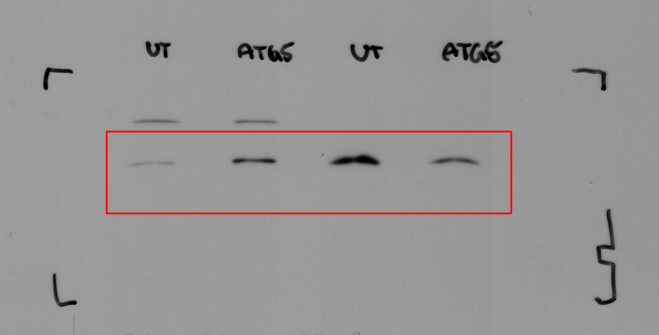

Supplement: Supplementary file 4 — Source Data [file 41467_2024_52875_MOESM4_ESM.zip › WB Full Scans/Figure_3/K/Fig.3_K_Sod1.tif]

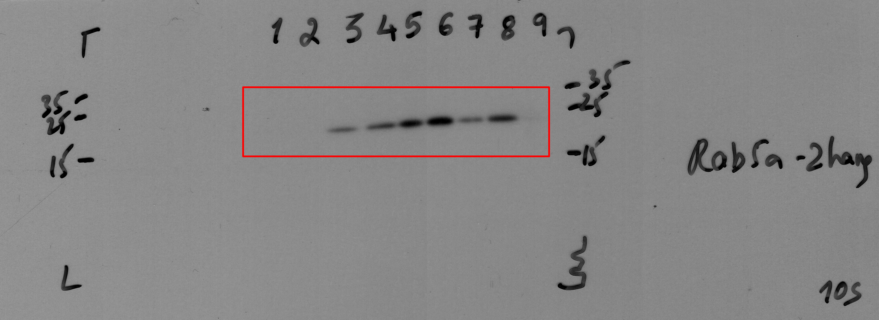

Supplement: Supplementary file 4 — Source Data [file 41467_2024_52875_MOESM4_ESM.zip › WB Full Scans/Figure_3/P/Fig.3_P_Rab5a.tif]

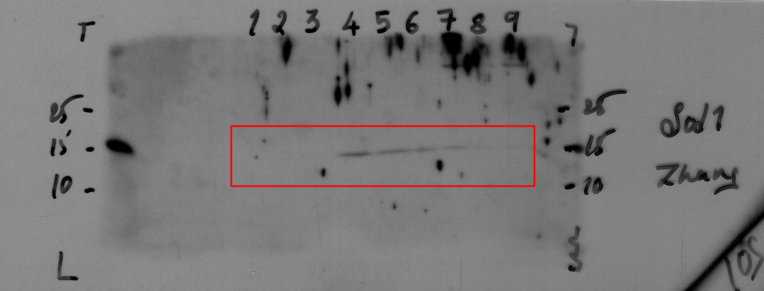

Supplement: Supplementary file 4 — Source Data [file 41467_2024_52875_MOESM4_ESM.zip › WB Full Scans/Figure_3/P/Fig.3_P_Sod1.tif]

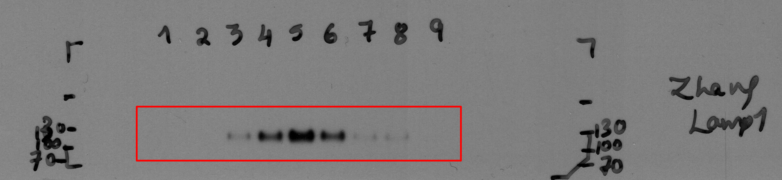

Supplement: Supplementary file 4 — Source Data [file 41467_2024_52875_MOESM4_ESM.zip › WB Full Scans/Figure_3/P/Fig.3_P_Lamp1.tif]

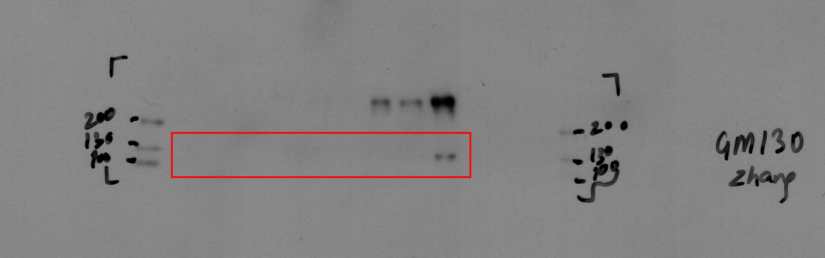

Supplement: Supplementary file 4 — Source Data [file 41467_2024_52875_MOESM4_ESM.zip › WB Full Scans/Figure_3/P/Fig.3_P_GM130.tif]

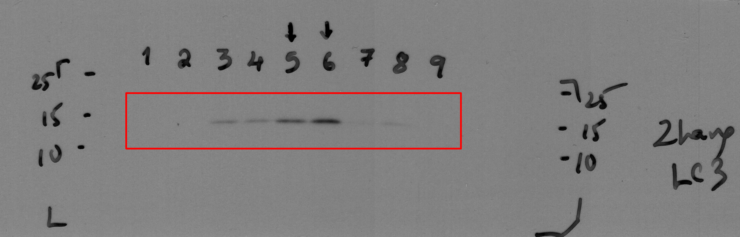

Supplement: Supplementary file 4 — Source Data [file 41467_2024_52875_MOESM4_ESM.zip › WB Full Scans/Figure_3/P/Fig.3_P_LC3.tif]

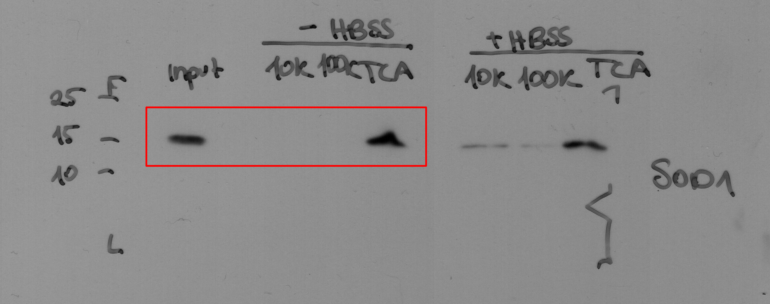

Supplement: Supplementary file 4 — Source Data [file 41467_2024_52875_MOESM4_ESM.zip › WB Full Scans/Figure_4/I/Fig.4_I_Sod1.tif]

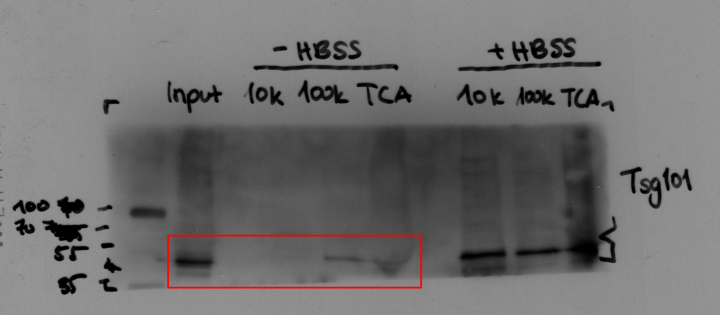

Supplement: Supplementary file 4 — Source Data [file 41467_2024_52875_MOESM4_ESM.zip › WB Full Scans/Figure_4/I/Fig.4_I_Tsg101.tif]

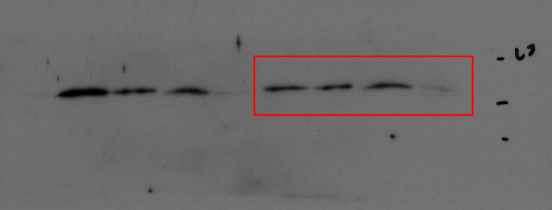

Supplement: Supplementary file 4 — Source Data [file 41467_2024_52875_MOESM4_ESM.zip › WB Full Scans/Figure_4/N/Fig.4_N_Sod1.tif]

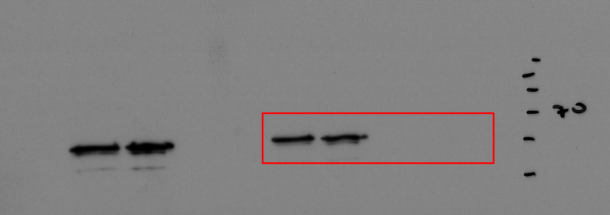

Supplement: Supplementary file 4 — Source Data [file 41467_2024_52875_MOESM4_ESM.zip › WB Full Scans/Figure_4/N/Fig.4_N_Tuj1.tif]

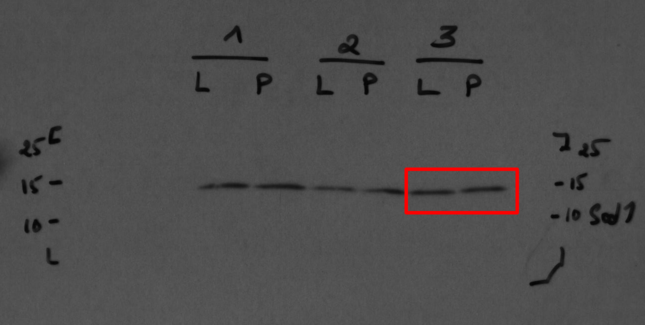

Supplement: Supplementary file 4 — Source Data [file 41467_2024_52875_MOESM4_ESM.zip › WB Full Scans/Figure_4/A/Fig.4_A_Cytosol_SOD1.tif]

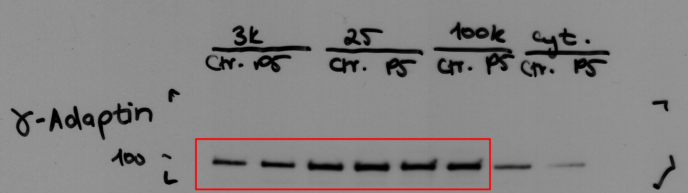

Supplement: Supplementary file 4 — Source Data [file 41467_2024_52875_MOESM4_ESM.zip › WB Full Scans/Figure_4/A/Fig.4_A_25k_y-Adaptin.tif]

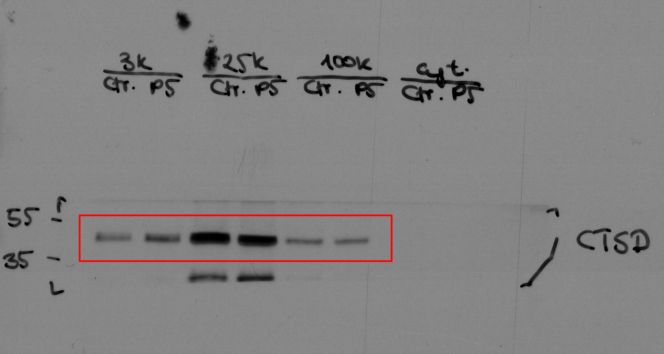

Supplement: Supplementary file 4 — Source Data [file 41467_2024_52875_MOESM4_ESM.zip › WB Full Scans/Figure_4/A/Fig.4_A_25k_Cathepsin-D.tif]

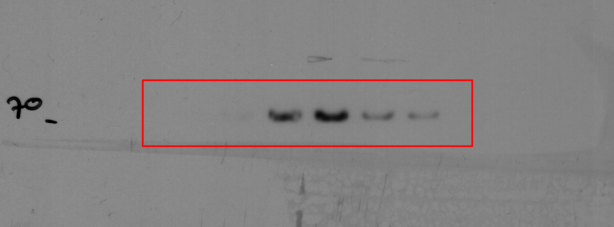

Supplement: Supplementary file 4 — Source Data [file 41467_2024_52875_MOESM4_ESM.zip › WB Full Scans/Figure_4/A/Fig.4_A_25k_Lamp1.tif]

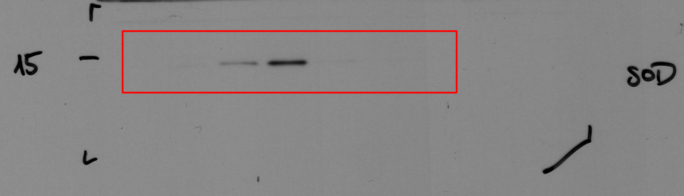

Supplement: Supplementary file 4 — Source Data [file 41467_2024_52875_MOESM4_ESM.zip › WB Full Scans/Figure_4/A/Fig.4_A_25k_SOD1.tif]
